# The Health Effects of Real-World Dual Use of Electronic and Conventional Cigarettes versus the Health Effects of Exclusive Smoking of Conventional Cigarettes: A Systematic Review

**DOI:** 10.3390/ijerph192013687

**Published:** 2022-10-21

**Authors:** Charlotta Pisinger, Sofie K. Bergman Rasmussen

**Affiliations:** 1 Center for Clinical Research and Prevention, Bispebjerg-Frederiksberg University Hospital, Capital Region of Denmark, 2400 Copenhagen, Denmark; 2 Danish Heart Foundation, 1120 Copenhagen, Denmark; 3 Department of Public Health, Faculty of Health and Medical Sciences, University of Copenhagen, 1165 Copenhagen, Denmark

**Keywords:** e-cigarettes, electronic cigarettes, ENDS, vaping, cigarettes, smoking, dual use, health, public health, biomarker

## Abstract

Background: A high prevalence of dual use of e-cigarettes and conventional cigarettes has been reported across the world. Methods: A systematic search was carried out. We included original articles on any topic relevant to health, excluding mental health, in all languages. The PRISMA guidelines were followed. Both reviewers independently screened and read all publications. We compared dual use with exclusive smoking of conventional cigarettes (ESCC). Results: Fifty-two publications (49 studies) were included. Thirteen papers/10 studies were prospective. There was great heterogeneity across studies. Many methodological weaknesses, such as inaccurate exposure measurement, lack of adjustment for former tobacco consumption, and lack of significance testing were identified. Most prospective studies found dual use to be at least as harmful as ESCC. The longest follow-up was six years. Most of the best available cross-sectional studies found dual use associated with the same and, in several studies, significantly higher risk of self-reported symptoms/disease than in ESCC. The intensity of cigarette smoking seems associated with worse health. Conclusion: Existing studies indicate that dual use is at least as, or probably even more, harmful than ESCC. Due to the predominance of cross-sectional studies and the methodological weaknesses we judged the overall certainty of the evidence as “low certainty”.

## 1. Introduction

The number of electronic cigarette/e-cigarette (EC/ECs) users has been increasing rapidly, and in 2021 it was estimated that there were 82 million vapers worldwide [1].

Available evidence on the benefits and risks of EC use are mixed and interpreted differently. Some believe that ECs have the potential to reduce the burden of disease in smokers [2,3] while others worry about the impact on public health and do not recommend, and even ban, their use [4,5]. Debates about the population health impact of alternative nicotine delivery products (i.e., ECs) are ongoing [6]. Public health research can be helpful by providing the needed evidence and facilitating its interpretation in a well-framed decision context.

A very important common reason for EC use in adults is smoking cessation [7,8] and ECs are recommended as cessation tools in some countries [9,10]. Smokers often buy ECs because they want to stop smoking, and though many succeed in switching to ECs for a short period, most relapse to conventional cigarette smoking [11]. The use of ECs and conventional cigarettes at the same time (CC/CCs) is called *dual use* (DU) [12,13,14,15,16]. A review found that dual users (DUs) perceive ECs as a safer and less addictive alternative to CCs [17].

Several studies across the world have reported a high prevalence of DU [12,13,14,15,18,19,20]. Studies from South Korea found DU in almost all current EC users [21,22,23]. A huge population-based study from the United States of America (USA) reported that more than six out of ten EC users were DUs [24], corresponding to approximately 1–3% of the population [12,22,24,25,26,27]. In contrast, some countries have lower estimates. A large population-based study from the United Kingdom (UK) found that less than 40% of adult EC users were DUs [25].

Dual use might be a short transition period before quitting smoking completely. However, a cohort study with six years of follow-up found that most DUs relapse to exclusive smoking of conventional cigarettes (ESCC) and only few transition exclusively to EC use [28,29]. Several other studies have reported the same similar findings [30,31,32,33,34].

A further concern is that there may not be a significant reduction in DUs’ consumption of CCs [35,36,37]. For example, a study from Poland found that DUs did not smoke fewer conventional cigarettes per day (CPD) than ESCC [38].

While health effects of smoking as well as vaping have been extensively studied, it is also important to understand the health effects of DU, as inhalation of smoke and EC aerosol in combination, in the worst case, could potentially lead to higher pathology than either inhalant alone. To our knowledge, only one review has investigated the health effects of DU (in pregnancy) [39]. The aim of this systematic review was to gather the existing evidence comparing the health effects of DU with the health effects of ESCC.

## 2. Materials and Methods

We followed the Preferred Reporting Items for Systematic Reviews and Meta-analyses (PRISMA-2020) guidelines.

Common components of ECs include a battery, heating coil, atomizer that transforms the e-liquid to an aerosol, cartridge that contains the e-liquid, and mouthpiece. Each component has the potential to affect health outcomes independently. The design of ECs has evolved in several ways since their introduction [40,41]. We included all types and all four generations of ECs.

### 2.1. Eligibility Criteria

Original articles on real-world DU of ECs and CCs on any topic relevant to health in any language.

### 2.2. Exclusion Criteria

Several cross-sectional studies have investigated the association between mental health problems and DU. These studies were not included, as we found it difficult to distinguish between mental health problems being a result of DU or a predictor of DU. Conference abstracts and dissertations were not included. Animal and human short-term experimental studies with forced switch or forced reduction in number of CPD were not included, as these studies do not reflect real-world use.

### 2.3. Information Sources

A search was carried out in PubMed, EMBASE, CINAHL, and Cochrane library (Table S1).

### 2.4. Search Strategy

The first search was conducted on 12 January 2021. The last search was conducted on 27 April 2021. We used the keywords “dual use” AND “e-cigarette” OR “e-cigarettes” OR “electronic cigarette” OR “electrically heated cigarette” OR “electronic nicotine delivery system” OR “electronic nicotine delivery device.” Keywords had to be included in the title, abstract, and/or full text. We used no other filters or limits. 

### 2.5. Selection Process

**Figure 1:**
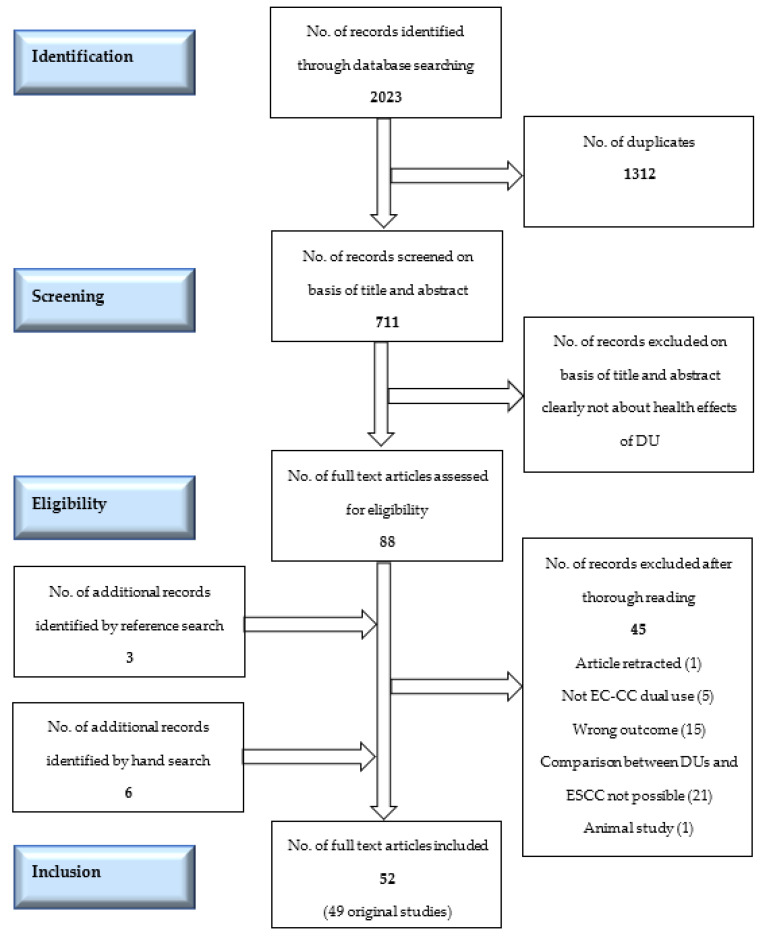
The PRISMA flow-chart of the search and inclusion of the papers in the systematic review. DUs= dual users. CC= conventional cigarettes. EC= e-cigarettes. ESCC = exclusive smokers of conventional cigarettes.

The screening process was blinded (Covidence was used for screening), and agreement of both authors was necessary to include/exclude a title. Eighty-eight papers were assessed for eligibility (Figure 1). All were in English. An overview table of all excluded papers can be found in Table S2. Additionally, references from the screened full text papers were carefully examined for missed papers, and our own reference data base was hand-searched for possible overlooked titles. A total of 52 papers (49 studies) were included in the final analysis.

### 2.6. Data Collection Process

During the data collection process, each reviewer independently read the full paper and extracted data from each paper to a predefined table framework. Results from the independent data collection were then compared, discussed, and merged into one detailed table (Table S3). We did not obtain or confirm data from study investigators.

### 2.7. Risk of Bias and Quality Assessment

As studies used very heterogenic methods and exposure measurement, it was not possible to use a universal assessment method. Prospective studies were assessed by the JBI critical appraisal tool (Appendix S1 in Supplementary Materials). Both reviewers independently assessed the main risks of bias without the use of automated tools. We registered conflict of interest (COI) and the size of the study and looked to see whether data were weighted for non-participation and if the study had taken relevant confounding into account. We further registered if studies had adjusted for former tobacco consumption. When assessing selection bias, we considered sampling, volunteer bias, and attrition bias. If several/many studies had investigated the same outcome, best quality studies were defined as “best available.”

### 2.8. Effect Measures

Effect measures varied depending on the outcome. Most papers on symptoms or disease risk presented unadjusted and adjusted odds ratios (OR) with a 95% confidence interval (CI). If available, we present adjusted odds ratios (aOR, Appendix S3 in Supplementary Materials). Papers on toxic effects typically presented geometric means and 95% CI and/or ranges and interquartile intervals.

### 2.9. Data Items 

Papers were included if any outcome data comparing DU with ESCC were presented, even if significance levels between ESCC and DU were not shown. We extracted the same predefined information from all papers (Table S3). If data on any variable were missing, we searched in Supplementary Materials and/or in study protocols.

### 2.10. Synthesis Methods

There was great heterogeneity both in exposure, methods, and outcomes across papers, so merging of results in a meta-analysis was not possible. After completing Table S3, which gave us an overview of all studies, we distributed papers according to two categories: prospective studies (Table 1) and cross-sectional studies (Table 2). Results were synthesized into five overall categories, marked by signs (##; #; ¤; *; **).

## 3. Results

### 3.1. Study Design

Table S3 shows the characteristics of the studies, study design, definition of use, and prevalence of use in detail. Most papers (75%) presented results of cross-sectional analyses based on self-reported data from large population-based surveys. Thirteen papers/10 studies reported results from studies with a prospective design (Table 1).

### 3.2. Definitions of Use (Exposure Measurement)

Exposure measurement was inaccurate in most studies (Appendix S2 in Supplementary Materials) and most papers did not specify the type of EC device or EC-fluid used. The definitions of ESCC, EC users, and DUs were self-reported. Duration and frequency of use of products varied widely. The criteria for current use were “use in the past 30 days” in many studies. Several studies defined smokers and EC users differently, and some had complex definitions. A study including adolescents defined users as “ever users” [62]. Many studies combined daily and non-daily use. Data on the duration of EC use was not presented in most studies. Only two cross-sectional studies distinguished between occasional and daily use [26,64].

### 3.3. Conflict of Interest

Fourteen (27%) of the papers had a COI: two with an EC manufacturer [52,56], one with the tobacco industry [29], two had received financial support from anonymous contributors [29,50], and the remaining had a COI with pharmaceutical companies.

### 3.4. Quality Assessment

Overall, 13 of 52 papers (one in four), were rated as having a high risk of selection bias. Only seven papers (five of these were prospective)/three studies had taken former tobacco consumption into account [28,29,35,44,50,58,69]. The overall quality of the prospective studies was generally high, as assessed by the JBI tool (Appendix S1 in Supplementary Materials), except for general inaccurate exposure measurement and lack of adjustment for former tobacco consumption (eight studies). Most of the cross-sectional studies were large, representative of the general population, had weighted data, and had a low risk of bias [26,64]. The primary aim of many studies was to compare EC users with smokers or non-users of tobacco or nicotine products, so they did not test for significance between DU and smokers.

### 3.5. Findings from the Prospective Studies (Table 1)

Four good-quality pregnancy risk assessment studies investigated DUs’ risk, but significance levels were not tested. Two studies reported that DUs had higher odds of giving birth to a small-for-gestational-age child than ESCC [42,43] and one found higher incidence of birthweight (<10th percentile) and a higher rate of admission to a neonatal intensive care unit in offspring of DUs than in offspring of ESCC [45]. However, the latter study also found the same birthweight, Apgar score, and gestation at delivery in offspring of DUs as in offspring of ESCC [45], and another study found that DUs had the same (elevated) risk of small-for-gestational-age and preterm birth as ESCC [46]. Furthermore, a very large cohort study of young women found a lower but not significantly different fecundability ratio in DUs than in ESCC [44].

Four good-quality papers described results from a cohort study with the longest follow-up that included almost 1400 persons at baseline [29]. There was a low drop-out rate during the six years and the study adjusted for former tobacco consumption and other relevant confounders and used advanced analyses. After one year, DUs had the same self-reported health as ESCC [35]. Two years after baseline, DUs still had the same self-reported health as ESCC and a significantly higher probability of serious adverse events [28]. However, six out of ten DUs stopped using ECs and continued to smoke CCs; those who still were DUs at the 24-month follow-up had significant improvement in self-rated health. After four years, there was no significant difference in self-reported health score and possible smoking-related disease between the DU group and CC users, but the study found generally worse outcomes in DUs [50]. After six years, self-reported health showed a very small change over time in all smoking status groups. Dual users’ risk of a possibly smoking-related disease did not differ significantly from ESCC (aOR of 1.48, 95% CI 0.81–2.70). The results did not differ substantially when the sample was restricted to those who did not switch smoking/vaping group or to those who had their outcomes confirmed through a linkage with hospital discharge abstracts [29].

Two very large, good-quality cohorts investigated pulmonary effects. One study found higher odds of self-reported respiratory disease in DUs [48] and the other found a higher incident rate of acute respiratory infections than in ESCC [49], but significance levels were not tested. A large nationally representative cohort of adolescents found higher, but not statistically significant, risk of sleep-related complaints than ESCC [51].

Thus, all these cohorts, of good quality, found the same or higher risk of negative health outcomes in DUs than in ESCC. This is in contrast with a small smoking cessation study with four weeks follow-up that found reductions in harmful substances in DUs after switching from ESCC [47].

### 3.6. Cross-Sectional Studies (Table 2)

Ten studies investigated the biomarker levels of harmful and potentially harmful substances (such as tobacco-specific nitrosamines, benzene, metals, or volatile organic compounds) in urine, blood, hair, and saliva of DUs and ESCC. Results of the two largest, best available, nationally representative studies found significantly higher biomarker levels of most of the measured 50 harmful substances [15] and of several toxic and carcinogenic substances [57] in DUs compared to ESCC. Another large, good-quality study found the same biomarker levels of harmful substances [54].

Most of the small studies (six had either a high risk of selection bias and/or lack of adjustment for confounders) found that levels of harmful substances were significantly lower or the same in DUs as in ESCC [52,53,55,56,59]; however, one found that DUs had higher levels for eight out of 11 metals tested [52]. Another study, with high risk of selection bias but adjustment for former tobacco consumption, included volunteers with long-term use of ECs and found that DUs had similar levels of most harmful substances but a significantly higher level of benzene (carcinogenic) than ESCC [58].

Eleven large good-quality surveys (eight of these with low risk of selection bias, weighted data, and adjusted analyses), including between eight and >700,000 persons from the general population, investigated the association between DU and self-reported respiratory symptoms/disease. Most of the surveys found a little higher/or the same (not significant/significance not tested) [12,60,61,63,65,68] odds of asthma or respiratory symptoms in DUs compared with ESCC, or significantly higher odds [66]. One of the surveys, including adolescents, found lower odds of asthma in DUs compared with ESCC, but significance level was not tested [61].

Three good-quality surveys investigated risk of self-reported chronic obstructive pulmonary disorder (COPD) and found significantly higher odds [64] and higher but not significant odds in DUs than in ESCC [67,68].

Ten large, good-quality surveys, including between 3400 and almost 450,00 persons from the general population, investigated the cardiovascular (CVD) and metabolic health effects of DU. The best available of these studies had adjusted for tobacco consumption and found higher HbA1c levels in DUs than in ESCC, but significance levels were not tested [69]. Four of the good-quality surveys investigated cardiovascular risk factors and found that DUs had a significantly higher OR of cardiovascular disease [26], significantly higher prevalence OR of cardiovascular risk factors and diagnosis of metabolic syndrome [21], significantly higher OR of elevated human c-reactive protein (CRP) [71], significantly higher risk of stroke [73], significantly higher prevalence of arrythmia [66], significantly higher OR of elevated CRP [71], and significantly higher OR of abdominal obesity than ESCC [22]. The two remaining surveys found higher OR of myocardial infarction and stroke, but significance level was not tested [65,74], and higher but not significant OR of hypertension [27] in DUs than in ESCC. Furthermore, one survey found that DUs had similar fasting glucose as ESCC [21], and another study found the same levels of insulin resistance [72].

A clinical study performed vascular function testing in almost 500 young persons, and reported that DUs had similar arterial stiffness as ESCC [70]. Eight cross-sectional studies investigated other health outcomes. Large good-quality surveys (only one did not weight data) including adults found that DUs had significantly worse fitness [76] and significantly higher levels of uric acid and prevalence of hyperuricemia [23] compared with ESCC. Further, higher odds of COVID-19 symptoms and higher odds of confirmed/suspected COVID-19 diagnosis were found in DUs [25,77] than in ESCC, but significance levels were not tested. Large surveys including adolescent DUs reported insufficient sleep significantly more often than ESCC [79], and higher odds of dental problems, but significance level was not tested [75]. In a large survey, homeless persons with DU reported significantly higher rates of asthma and cancer compared to ESCC [78]. Finally, in a small human clinical study, DUs had higher levels of most biomarkers of systemic inflammation than ESCC, but the difference was not significant [80].

### 3.7. Intensity of Smoking or EC use and Impact on Health

Most of the studies did not collect robust data on the level of EC and CC consumption. Those that did, found that DUs smoked the same [15,21,29,35,57,76] or a significantly higher number of CPD as ESCC [66].

A smoking cessation study showed a correlation between the number of CPD and harm in DUs. DUs who had been able to reduce the number of CPD substantially had reduced biomarker levels [47]. Moreover, one of the large, best available surveys showed that the frequency of CC use was positively associated with toxicant concentration [15]. Most of the studies that reported the same or higher tobacco consumption in DUs as in ESCC found significantly worse health outcomes in DUs than in ESCC [15,21,29,57,66,76]. None of the studies where DUs reported smoking a lower number of CPD than ESCC found a significantly worse outcome in DUs [52,59,62]; in fact, one of them found that ESCC had a worse outcome [59].

Only two cross-sectional studies investigated the potential impact of the frequency/dose of EC use by DUs. A survey of good quality found that the risk of premature CVD was significantly higher in DUs with a daily use of ECs than in those with occasional use of ECs [26]. Another survey of good quality found higher OR of COPD with increasing frequency of EC use among people who had never smoked, indicating a stepped harm of EC use [64].

### 3.8. Synthesis of the Results

Based on a very limited number of prospective studies, DU seems to be at least as harmful as ESCC. The same tendency was found in large, best available cross-sectional studies that found DU associated with the same, and in several studies significantly higher, risk of self-reported symptoms/disease as ESCC and in the largest population-based best available studies that found lower levels of harmful substances ESCC than in DUs. The intensity of smoking seems associated with worse health outcomes in DUs but very few studies investigated this.

Due to the predominance of cross-sectional studies, the inaccurate exposure measurement, and a high risk of reverse causality, we judged the overall certainty of the evidence in this review as “low certainty.”

## 4. Discussion

### 4.1. Overall Findings

This is the first systematic review comparing the general health effects of real-world DU with ESCC. We identified 52 papers/49 studies. Only one of four studies had a prospective design. There was great heterogeneity across studies, both in the definition of use, in methodology, and in outcome measurement, so only a narrative review was possible. Many studies did not test for significance. The best available studies found a tendency to at least the same or higher levels of harmful substances, and at least the same or higher risk of harmful effects in DUs compared with ESCC. Due to the predominance of cross-sectional studies, the inaccurate exposure measurement and high risk of reverse causality, the evidence in this review is rated as “low certainty.”

### 4.2. Comparison with Other Studies and Considerations about Findings

If smokers replaced all/most of the CCs with ECs, there might be a beneficial effect of DU [81]. Short-term experimental studies of forced switch from CCs to ECs have shown reduced levels of harmful substances in DUs compared with ESCC [82], and the degree of reduction to be proportional to the reduced numbers of CPD. However, the “real-world use” studies included in our review found that DUs and ESCC smoked the same amount of tobacco/number of CPD, and one study even found a significantly higher number of CPD in DUs than in ESCC [66]. The lack of significant reduction in number of CPD in DUs agrees with other studies not included in this review [35,36,37,83,84]. The intensity of smoking in DUs was (not surprisingly) found to be associated with worse health outcomes, but the majority of the studies included in the review did not collect robust data on the level of CC consumption.

If DUs do not reduce the number of CPD but use ECs as a supplement, the combination of CCs and ECs might potentially be even more harmful for health than ESCC, as known and unknown [85] harmful substances and transformation products formed by heating of ECs [86] are added on top of the harmful substances in tobacco smoke. The long-term effects of EC use on human health will take a long time to be fully elucidated, but concern has been raised [87,88,89,90,91]. Although several studies have found an increased risk of disease in EC users [48,66,87,92,92,93,94,95] and shown that, e.g., the cardiovascular [96] and pulmonary harm from ECs is biologically plausible [88], at present the risk in the human population is uncertain [81,97].

It was not possible to distinguish the root of a health event/outcome/disease (i.e., caused by tobacco use, or ECs, or a combination), as few studies measured intensity of smoking and even fewer the intensity of EC use. The intensity of EC use was only investigated in two cross-sectional studies, so it is impossible to establish a dose-dependent relationship. Furthermore, the evolving design of the EC devices [98] and the huge heterogeneity of the content of EC-fluid might both have an impact on DU frequency in the population and on the health-related risk. We need further investigation of the impact of dose, frequency, and duration of EC use, as well as the potential biological interactions of ECs and CCs.

We cannot draw conclusions on causality from cross-sectional studies. Moreover, most DUs have been ESCC for decades and might already have had a smoking-related disease before they started vaping. In some studies participants were included if they had *ever* been diagnosed with, for example, heart disease [26] or stroke [73], but there was no information on whether they had the disease before or after they started using ECs. The prospective study by Bhatta found reverse causality *p* < 0.001 [48] but only seven papers/four studies adjusted for differences in previous tobacco consumption. The risk of reverse causality is probably the greatest challenge. An Italian good-quality cohort study with six years follow-up, however, had adjusted for duration of tobacco smoking. The study found that the risk of possible smoking-related disease was slightly, but not significantly, worse in persistent DUs [29].

Moreover, longer follow-ups are required. In a sample comprised of former smokers, a decade or more would be more appropriate to detect significant risk reductions.

Confounding factors could be another reason for the tendency towards worse health outcomes in DUs but most adjusted for relevant factors, such as exposure to second-hand smoke, and almost all adjusted for sociodemographic factors [61]. Few of the included studies were designed to compare DUs with ESCC and many did not test for significance.

Misclassification might also be a problem regarding smoking/vaping status [87] and the definition of DU also varied a lot across studies. Disease was mostly self-reported, which imposes the risk of both recall and misclassification bias. However, a large retrospective survey using hospital records found a tendency towards a higher risk of respiratory infections in DUs [49].

Conversely, the included studies had also several strengths. The prospective studies were of good quality, except for the exposure measurement, which was mostly inaccurate, and lack of adjustment for former tobacco consumption in most studies. Most of the cross-sectional studies were very large, good-quality population-based studies, reflecting real-world use in a general population. An example of an excellent study (providing the United States Food & Drug Administration (FDA) with detailed information) is the large, nationally representative longitudinal PATH study [99]. Moreover, even though most studies were cross-sectional, many environmental and occupational diseases had been detected first by cross-sectional approaches, long before cohort studies and intervention studies were available. For respiratory disease, Bhatta & Glantz showed a good agreement between longitudinal and cross-sectional results on ECs [48].

Tobacco regulation is a dynamic field. Health authorities rely on a robust science base to develop regulations that improve public health. Well-designed, large prospective studies on health effects of DU, considering not only sociodemographic confounders, but also age at onset of smoking, pack-years of CCs, and, e.g., alcohol consumption, are needed. Furthermore, exposure of CCs and ECs needs to be measured in a more detailed way, so a dose response can be investigated. For ECs, exposure measurement should include type of device, concentration of nicotine used, and duration, frequency (e.g., daily/weekly/monthly use), and intensity of vaping. Measuring vaping intensity is challenging. Puff topography, number of use sessions, and amount of EC liquid consumed are being used in some studies, but all these measures have limitations and development of uniform EC-use intensity measures is needed [100].

Exposure conditions that are relevant to real-world inhalation exposure in humans should be used in human and animal experimental models. The National Academies of Sciences, Engineering, and Medicine (NASEM) report on ECs provides a thorough list of research needs [81].

### 4.3. Limitations of the Review

Due to the large heterogeneity (methods, exposure, outcomes) of the studies, we were unable to assess study quality in a systematic way nor were we able to perform a meta-analysis. We decided to include all studies, independent of study design and the year of publication. Due to the huge evolution of the EC devices, we might have restricted our search to newer studies, but only four studies were more than five years old (at the time of our search), so the devices used were generally up to date. We could also have restricted our search to prospective studies only but chose to describe if studies were “best available”/had good quality. Moreover, this review did not explore the potential health benefits of DU in smokers who reduce their CPD by, e.g., 50% or more by use of ECs. Many outcomes did not test for significance; therefore, the increased ORs reported in these studies may not indicate a significant increase in adverse outcomes compared to ESCC use. We could have decided not to include studies without a test for significance but chose to include them and show odds ratios in Appendix S3 in Supplementary Materials.

Interpretation of our findings for DUs was challenging because of heterogeneity of the DU group, which can include both habitual heavy smokers who occasionally vape and heavy vapers who only smoke a little. We might have included the experimental forced-switch/CPD reduction studies but they do not reflect real-world use. We might also have excluded the experimental smoking cessation study with very short-term follow-up [47], but smokers could use ECs ad libitum, which reflects real-world use. Furthermore, we might have been more critical regarding the validity of outcomes, such as self-reported symptoms. Finally, an interpretation bias must always be kept in mind.

### 4.4. Strengths of the Review

Both authors independently read all abstracts and full text of papers and extracted data. Eventual disagreements were discussed. Potential COIs that might influence the findings were described. Quality assessment was performed, even though it could have been more thorough. This review gives a real-world picture of health effects of DU in the general population.

## 5. Conclusions

A high prevalence of DU has been reported across the world. In some countries, most users of ECs are DUs. This is the first systematic review comparing the health effects of DU with ESCC. Based on a very limited number of prospective studies, DU seems to be at least as, or probably even more harmful than, ESCC. The same tendency was found in cross-sectional studies; several of the best available studies found the same or higher risk of symptoms/disease or level of harmful substances in DUs than in ESCC. The intensity of smoking seems associated with worse health outcomes but very few studies investigated this. Due to the predominance of cross-sectional studies, the inaccurate exposure measurement, and a high risk of reverse causality, we judged the overall certainty of the evidence in this review as “low certainty.” Well-designed prospective studies on health effects of DU are needed.

## Figures and Tables

**Table 1:** Prospective studies investigating the potential harm in DUs compared to ESCC.

	First Author, Year of Publication, Country, Conflict of Interest	Method	Participants	Risk of Selection Bias/Weighted Data/Adjusted Analyses/Adjusted for Former Tobacco Consumption	Major Outcomes	Overall Findings Significantly (Higher Risk/Prevalence/Level in ESCC = ## Higher Risk/Prevalence/Level in ESCC, Significance Level Not Tested or Not Significant = # Same Risk/Prevalence/Level in ESCC and DUs = ¤ Higher Risk/Prevalence/Level in DUs, Significance Level Not Tested or Not Significant = * Significantly Higher Risk/Prevalence/Level in DUs = **)
*Pregnancy and fertility*	Cardenas V.M. [42] 2020, USA None	Pregnancy Risk Assessment Monitoring	1594 pregnant women	Low/yes/yes/no	Risk of small-for-gestational-age	DUs: higher odds of giving birth to a small-for-gestational-age child than ESCC, but significance level not tested *
	Clemens M.M. [43] § 2019, USA None	Pregnancy Risk Assessment Monitoring	248 pregnant women	Low/yes/yes/no	Carcinogen metabolites (TSNAs) in hair samples + Risk of small-for-gestational-age (SGA)	DUs same level of carcinogen biomarkers as ESCC ¤ DUs had higher risk of small-for-gestational-age than ESCC, but significance not tested *
	Harlow A. [44] 2020, USA None	Cohort study, online survey	4586 young women trying to conceive	Low/no/yes/ yes	Fecundability (menstrual cycle and achieved pregnancy)	DUs: lower fecundability ratio than ESCC, but not significantly different *
	McDonnell BP. [45] 2020, Ireland None	Pregnancy Risk Assessment Monitoring	620 pregnant women	Low/no/yes/no	Delivery and neonatal outcomes	DUs: same birthweight, Apgar score and mean gestation at delivery as ESCC ¤ DUs: higher rate of admission to neonatal intensive care unit and higher incidence of birthweight <10th centile than ESCC but significance level not tested *
	Wang X. [46] 2020, USA None	Pregnancy Risk Assessment Monitoring	31,973 pregnant women	Low/yes/yes/no	Preterm birth and small-for-gestational-age (SGA)	Similar (elevated) risk of preterm birth and of small-for-gestational-age in DU as ESCC ¤
*Other*	McRobbie H. [47] 2015, UK 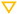 Yes	Smoking cessation study with 4 weeks follow-up	44 healthy volunteer smokers Use of EC ad libitum	High/-/no/no	Urinary 3-HPMA, a major metabolite of acrolein and carbon-monoxide (CO)	DUs had sign. reductions in 3-HPMA and CO after switching from ESCC (significant reduction in cotinine in DU) ##
	Bhatta D. N. [48] 2020, USA None	Nationally representative cohort study	32,320 adults	Low/yes/yes/no	Self-reported respiratory disease (chronic obstructive pulmonary Disease (COPD), chronic bronchitis, emphysema, or asthma)	DUs: higher odds of reporting of respiratory disease than ESCC but significance level not tested *
	Sanou A. Z. [49] 2020, USA None	Register study using a cohort	802,621 adult military members	Low/no/yes/no	Incident cases of acute respiratory infections (in- and outpatient diagnoses)	DUs had higher incident rate of acute respiratory infections than ESCC but significance level not tested *
	Flacco M. E. [50] ^^ 2019, Italy 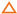 Yes	Cohort study 48 months	915 adults	High/no/yes/yes	Changes in self-reported health score and possibly smoking-related disease	DUs: no significant difference in self-reported health score and possible smoking related disease after 4 years than ESCC, but generally worse outcomes in DUs ¤
	Flacco M. E. [29] ^^ 2020, Italy 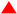 Yes	Cohort study 72 months	912 adults	High/no/yes/yes	Changes in self-reported health score and possibly smoking-related disease	DUs had higher odds of possibly smoking related disease after 6 years than ESCC but not significant ¤
	Manzoli L. [35] ^^ 2015, Italy None, first 2 years	Cohort study 12 months	959 adults with 1-year data	High/no/yes/yes	Self-reported health	DUs: same self-reported health as ESCC ¤
	Manzoli L. [28] ^^ 2017, Italy None, first 2 years	Cohort study 24 months	932 adults with 2-year data	High/no/yes/yes	Self-reported health	DUs at baseline: same self-rated health as ESCC and significantly higher probability of serious adverse events than ESCC ¤ ** DUs at 24 months follow-up: significant improvement in self-rated health compared with ESCC ##
	Riehm K. E. [51] 2019, USA 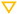 Yes	Nationally representative cohort	9588 adolescents	Low/yes/yes/no	Sleep-related complaints	DUs: higher risk of sleep-related complaints than ESCC, but not significant *

^^ Same cohort: [28,29,42,52]. § Pregnancy + other outcomes. 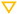 Conflict of interest: pharmaceutical industry; 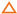 Conflict of interest: financial support from anonymous contributors; 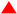 Conflict of interest with the tobacco or e-cigarette industry.

**Table 2:** Cross-sectional studies investigating potential harm of DUs compared to ESCC.

	First Author, Year of Publication, Country, Conflict 0f Interest	Method	Participants	Risk of Selection Bias/Weighted Data/Adjusted Analyses/Adjusted for Former Tobacco Consumption	Major Outcomes	Overall Findings Significantly (Higher Risk/Prevalence/Level in ESCC = ## Higher Risk/Prevalence/Level in ESCC, Significance Level Not Tested or Not Significant = # Same Risk/Prevalence/Level in ESCC and DUs = ¤ Higher Risk/Prevalence/Level in DUs, Significance Level Not Tested or Not Significant = * Significantly Higher Risk/Prevalence/Level in DUs = **
*Harmful substances*	Carroll D.M. [53] 2018, USA None	Cross-sectional study	94 volunteer adults of American Indian descent	High/-/(no)/no	Carcinogen metabolite (NNAL) in urine	DUs same level of carcinogen biomarker as ESCC ¤
	Goniewicz M. [15] 2018, USA 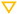 Yes	Cross-sectional analyses of nationally representative cohort study	5105 adults	Low/yes/yes/no	50 biomarkers of toxicity (TSNAs, metals, PAHs, and VOCs) in urine	DUs: significantly higher concentration of most biomarkers of toxicity/carcinogenicity than ESCC **
	Jain R. [54] 2019, USA None	Cross-sectional analyses of population-based survey	1139 adults	Low/yes/yes/no	Harmful metals (cadmium, lead, and mercury) in blood	DUs same levels of harmful metals in blood as ESCC ¤
	Keith R. [55] 2020, USA None	Cross-sectional analysis of cohort study	371 volunteer adults	Low/no/yes/no	Volatile organic compound (VOC) metabolites in urine	DUs and ESCC had similar levels of most VOC metabolites, except four, which were significantly higher in ESCC than in DU ¤ ##
	Piper M [34] 2018, USA None	Cross-sectional analysis of cohort study	422 volunteer adults	Low/no/yes/no	Carcinogen metabolite (NNAL) in urine	DUs had significantly lower levels of NNAL than ESCC ##
	Prokopowicz A. [52] 2019, Poland 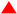 Yes	Cross-sectional study	156 young volunteer adults	High/-/yes/no	Harmful metals cadmium and lead in blood	DUs: levels of harmful metals not significantly different than ESCC ¤
	Prokopowicz A. [56] 2020, Poland 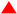 Yes	Cross-sectional study	88 young volunteer adults	High/-/yes/no	11 toxic metals in urine	Significance level between ESCC and DUs not tested, but DUs had higher values for 8 out of 11 metals in urine *
	Rostron B. L. [57] 2019, USA None	Cross-sectional analysis of a nationally representative cohort	2710 adults	Low/yes/yes/no	Carcinogen and toxin exposure, biomarkers (VOCs, PAHs and TSNAs) in urine and blood	DUs: significantly higher levels of some toxic and carcinogenic biomarkers (NNAL, 1-HOP, HPMA and MHB3) compared to ESCC ** ¤
	Shahab L. [58] 2017, UK 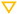 Yes	Cross-sectional study	181 volunteer adults with long-term use	High/-/yes/yes	Carcinogen and toxin exposure, biomarkers (VOCs and TSNAs) in urine and saliva	DUs and ESCC had similar levels of toxic and carcinogenic substances, but DU had significantly higher level of one carcinogenic substance, benzene than ESCC ¤ **
	Smith D. ^ [59] 2020, Poland, UK and USA 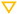 Yes	Cross-sectional study	456 volunteer adults with long-term use	High/-/yes/no	Carcinogen and toxin exposure biomarkers (VOCs, TSNAs and minor alkaloids) in urine and saliva	DUs and ESCC had similar levels of toxic and carcinogenic substances, but ESCC had significantly higher level of three TSNAs and acrylonitrile than DUs ¤ ##
	Cho J. H. [60] 2016, South Korea None	Nationally representative survey	35,904 adolescents	Low/no?/yes/no	Self-reported diagnosed with asthma	DUs higher odds of reporting asthma than ESCC but not significant in adjusted analyses *
	Chung S. J. [61] 2019, South Korea None	Nationally representative survey	60,040 adolescents	Low/yes/yes/no	Self-reported diagnosed with asthma or/and allergic rhinitis	DUs had higher odds for current allergic rhinitis but lower odds of current asthma than ESCC, but significance level not tested * #
	Hedman L. [12] 2018, Sweden 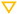 Yes	2 population-based surveys	30,272 adults	Low/no/yes/no	Self-reported respiratory symptoms: long-standing cough, sputum production, wheeze	DUs had higher odds of self-reported respiratory symptoms than than ESCC but significance level not tested *
	Lee A. [62] 2019, South Korea None	Population-based survey	58,336 adolescents	Low/yes/yes/no	Self-reported asthma, allergic rhinitis and atopic dermatitis	DUs har lower odds of asthma than ESCC, but comparable odds of allergic rhinitis and atopic dermatitis. Significance level not tested # ¤
	Li D. [63] § 2020, USA 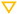 Yes	Nationally representative survey	28,171 adults	Low/yes/yes/no	Self-reported respiratory symptoms and physical health	DUs same odds of respiratory symptoms as ESCC ¤ DUs: same prevalence of poor physical health as ESCC ¤
	Osei A. [64] 2020, USA 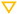 Yes	Nationally representative survey	705,159 adults	Low/yes/yes/no	Self-reported diagnosed with COPD/emphysema/chronic bronchitis	DUs had significantly higher odds of COPD/emphysema/chronic bronchitis than ESCC **
	Parekh T. [65] 2020, USA None	Nationally representative survey	161,965 young adult women	Low/yes/yes/no	Self-reported diagnosed with COPD/emphysema/chronic bronchitis and asthma	DUs had higher odds of asthma and COPD compared than ESCC, but significance level not tested *
	Wang J. B. [66] § 2018, USA 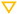 Yes	Internet population -based survey	39,747 adults	Low/no/yes/no	Self-reported cardiopulmonary symptoms in the last months General health in the last month (SF-12)	DUs had significantly higher/worse breathing difficulty score than ESCC ** DUs: significantly worse median general health scores than ESCC** DUs: significantly higher prevalence of history of an arrhythmia than ESCC **
	Wills T. A. [67] 2019, USA None	Population-based survey	8087 adults	Low/yes/yes/no	Self-reported diagnosed with asthma, COPD	DUs and ESCC same odds of asthma ¤ DUs higher odds of COPD than ESCC but not significant *
	Wills T.A. [68] 2020, USA None	Nationally representative youth survey	14,765 adolescents	Low/yes/yes/no	Self-reported asthma diagnosis	DUs had higher odds of asthma than ESCC but not sign. ¤ Significantly higher in a sensitivity analysis tested in a sample with complete data **
	Xie Z. [24] 2020, USA None	Nationally representative youth survey	887,182 adults	Low/yes/yes/no	Self-reported COPD diagnosis told by doctor	DUs had significantly higher risk of self-reported COPD diagnosis told by doctor than ESCC **
*Cardiovascular and metabolic outcomes*	Choi D-W [69] 2018, South Korea None	Nationally representative survey	8809 adults	Low/yes/yes/yes	Diabetes (HbA1c)	DUs had higher HbA1c levels than ESCC but significance level not tested *
	Fetterman J. [70] 2020, USA None	Human clinical study with noninvasive vascular function testing	467 younger adults	High/-/yes/no	Cardiovascular health (augmentation index)	DUs had similar arterial stiffness as ESCC ¤
	Kim C. [21] 2020, South Korea None	Population-based survey	7505 adult men	Low/yes/yes/no	Cardiovascular risk factors (waist circumference, blood pressure, triglycerides, fasting glucose, HDL-cholesterol, diagnosis of metabolic syndrome)	DUs had significantly higher prevalence odds ratio of cardiovascular risk factors (waist circumference, triglycerides, HDL-cholesterol, blood pressure) and diagnosis of metabolic syndrome than ESCC DUs had similar fasting glucose as ESCC ¤ **
	Kim T. [22] 2020, South Korea None	Nationally representative survey	14,738 adults	Low/yes/yes/no	Cardiovascular risk factors (waist circumference, blood pressure, triglycerides, fasting glucose, HDL-cholesterol, diagnosis of metabolic syndrome)	DUs had significantly higher odds of abdominal obesity than ESCC ** Other outcomes: no s significant difference but tendency to higher odds in DUs (except blood pressure) # *
	Mainous A. [71] 2020, USA None	Nationally representative survey	4659 adults	Low/yes/yes/no	Biomarker of inflammation and predictor of cardiovascular disease (CRP)	DUs had significantly higher odds of elevated CRP than ESCC **
	Miller C. R. [27] 2021, USA 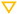 Yes	Population-based survey	19,147 adults	Low/yes/yes/no	Self-reported diagnosis of hypertension in the last 12 months	DUs had higher odds for hypertension than ESCC, but significance not reached (0.99 for lower 95%CI) *
	Orimoloye O. [72] 2019, USA None	Population-based survey	3415 adults	Low/yes/yes/no	Insulin resistance (measured by HOMA-IR and GTT levels)	DUs had same risk of insulin resistance as ESCC ¤
	Osei A. [26] 2019, USA None	Nationally representative survey	449,092 adults	Low/yes/yes/no	Self-reported diagnosed with cardiovascular disease (stroke, myocardial infarction or coronary heart disease)	DUs had significantly higher odds of CVD than ESCC ** DUs had significantly higher odds of premature CVD than ESCC **
	Parekh T. [73] 2019, USA None	Nationally representative survey	161,529 young adults	Low/yes/yes/no	Self-reported stroke	DUs had significantly higher risk of stroke than ESCC **
	Vindhyal M. [74] 2020, USA None	Nationally representative survey	16,855 adults	Low/yes/yes/no	Self-reported diagnosed with cardiovascular disease	DUs had higher odds of myocardial infarction and stroke than ESCC, but significantly level not tested *
*Other*	Akinkugbe A. A. [75] 2019, USA None	Population-based survey	13,650 adolescents	Low/yes/yes/no	Self-reported past-year diagnosis with dental problems	DUs: higher odds of dental problems than ESCC, but significance level not tested *
	Chen D. TH. [25] 2021, United Kingdom None	4 population-based surveys	13,077 adults	Low/yes/yes/no	Self-reported experience of COVID-19 symptoms and diagnosis	DUs had higher odds of covid-19 symptoms and higher odds of confirmed/suspected covid-19 diagnosis than ESCC but significance level not tested *
	Dinkeloo E. [76] 2019, USA None	Online survey	2854 men, soldiers	Low/no/yes/no	Physical activity	DUs: significantly worse fitness than ESCC **
	Gaiha S. M. [77] 2020, USA None	National online survey	4351 young adults	Low/yes/yes/no	Self-reported COVID-19 symptoms, testing and diagnosis	DUs higher risk of COVID-19 symptoms and diagnosis than ESCC but significance level not tested *
	Kim T. [23] 2021, South Korea None	Nationally representative population-based survey	10,692 adults	Low/yes/yes/no	Levels of serum uric acid and hyperuricemia	DUs significantly higher levels of uric acid and prevalence of hyperuricemia than ESCC **
	Leavens E. [78] 2020, USA None	Interview-survey	4148 homeless adults	High/no/no/no	Self-reported chronic health conditions	DUs significantly higher rates of asthma ** and cancer compared to ESCC **
	Merianos A. [79] 2021, USA None	School based nationally representative survey	11,296 high school students	Low/yes/yes/no	Self-reported duration of sleep	DUs were significantly more likely to report insufficient sleep compared with ESCC **
	Ye D. [80] 2020, USA None	Human clinical study	48 adults	High/-/no/no	Systemic inflammation, oxidative stress, angiogenesis and tissue injury/repair in saliva and gingival crevicular fluid (GCF)	DUs: higher levels of most biomarkers of systemic inflammation than ESCC, but no significant difference *

^ Participants from UK, Shahab 2017, also included in this study. § Also other health outcome than respiratory. 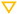 Conflict of interest: pharmaceutical industry. 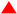 Conflict of interest with the tobacco or e-cigarette industry.

## Data Availability

Links of the databases used in the review: PubMed https://pubmed.ncbi.nlm.nih.gov/ (accessed on 10 October 2022), EMBASE https://www.embase.com/ (accessed on 10 October 2022), CINAHL https://www.ebsco.com/products/research-databases/cinahl-database (accessed on 10 October 2022) (local access), and Cochrane library https://www.cochranelibrary.com/search (accessed on 10 October 2022). All extracted data is available in the original papers; an overview of the extracted data is found in Table S3. Only a narrative review was performed, not a synthesis of data. An overview of excluded studies is available in Table S2.

## Supplementary Materials

The following supporting information can be downloaded at: https://www.mdpi.com/article/10.3390/ijerph192013687/s1 Table S1: Systematic search in databases; Table S2: Overview of excluded studies; Table S3: Detailed overview of the included studies; Appendix S1: JBI Checklist for cohort studies; Appendix S2: Definition and prevalence of use. Appendix S3: Characteristics of studies.

## Abbreviations

ESCC	exclusive smokers of conventional cigarettes
DU	dual use of conventional cigarettes and e-cigarettes
DUs	dual users of conventional cigarettes and e-cigarettes
COPD	chronic obstructive pulmonary disease
CRP	human c-reactive protein
CVD	cardiovascular disease
GCF	gingival crevicular fluid
3-HPMA	Urinary 3-hydroxypropyl mercapturic acid, a major metabolite of acrolein
TSNA	Tobacco-specific N-nitrosamines
PAH	polycyclic aromatic hydrocarbons
HDL	high density lipids
GTT	glucose tolerance test
HbA1c	glycosylated hemoglobin
HPMA	N-acetyl-S-3-hydroxypropylcysteine, a metabolite of acrolein (VOC)
MHB3	a metabolite of 1,3-butadiene (VOC)
NNAL	4-(methylnitrosamino)-1-(3-pyridyl)-1-butanol, the principal metabolite of the lung carcinogen NNK (TSNA)
PMA	benzene
VOC	Volatile organic compounds
