# Supplementary material for: The Health Effects of Real-World Dual Use of Electronic and Conventional Cigarettes versus the Health Effects of Exclusive Smoking of Conventional Cigarettes: A Systematic Review
Source: Int J Environ Res Public Health. 2022 Oct 21;19(20):13687. doi: 10.3390/ijerph192013687 (PMC9603628; doi:10.3390/ijerph192013687)
Supplement: Supplementary file 1 [file ijerph-19-13687-s001.zip › ijerph-1914366-supplementary.pdf]

Table S1. Systematic search in databases showing number of articles found (Number of searched articles/duplicates/included in screening process)

**First search:** all studies published before 12.01.2021									
	**PubMed**		**EMBASE**		**Cinahl**		**Cochrane**		Total number / duplicates / included in screening process
Search words	#	Number of searched articles	#	Number of searched articles	#	Number of searched articles	#	Number of searched articles	
(Dual use) AND (E-cigarette OR e-cigarettes)	1	493	2	199	3	231	4	43	966 / 406 / 560
**Second search:** all studies published before 27.04.2021									
	**PubMed**		**EMBASE**		**Cinahl**		**Cochrane**		Total number / duplicates / included in screening process
Search words	#	Number of searched articles	#	Number of searched articles	#	Number of searched articles	#	Number of searched articles	
(Dual use) AND (Electronic cigarette OR Electrically heated cigarette OR Electronic nicotine delivery system OR Electronic nicotine delivery device)	5	596	6	292	7	119	8	50	1.057 / 906 / 151
Found in searched articles									4 / 0 / 4
Searched by hand									6 / 0 / 6

Search number

Table S2. Overview of the 45 studies excluded in full text review. All studies are divided into six exclusion criteria-categories.

**Reference**	**Exclusion criteria**
Abafalvi L, Pénzes M, Urbán R, Foley KL, Kaán R, Kispélyi B, Hermann P: Perceived health effects of vaping among Hungarian adult e-cigarette-only and dual users: a cross-sectional internet survey. *BMC public health* 2019, 19(1):302.	Comparison between EC and CC not possible
Bhatta DN, Glantz SA: Electronic Cigarette Use and Myocardial Infarction Among Adults in the US Population Assessment of Tobacco and Health. *J Am Heart Assoc* 2019, 8(12):e012317.	Article retracted
Brożek GM, Jankowski M, Zejda JE: Acute respiratory responses to the use of e-cigarette: an intervention study. *Scientific reports* 2019, 9(1):6844.	Comparison between EC and CC not possible
Carroll DM, Wagener TL, Stephens LD, Brame LS, Thompson DM, Beebe LA: The relationship between nicotine metabolism and nicotine and carcinogen exposure among American Indian commercial cigarette smokers and electronic nicotine delivery system users. *Addict Behav* 2019, 92:58-63.	Wrong outcome
Cassidy RN, Tidey JW, Colby SM: Exclusive e-cigarette users report lower levels of respiratory symptoms relative to dual e-cigarette and cigarette users. *Nicotine Tob Res* 2020.	Comparison between EC and CC not possible
Czoli CD, Fong GT, Goniewicz ML, Hammond D: Biomarkers of Exposure Among"Dual Users" of Tobacco Cigarettes and Electronic Cigarettes in Canada. *Nicotine Tob Res* 2019, 21(9):1259-1266.	Not real-world study
Doran N, Brikmanis K, Petersen A, Delucchi K, Al-Delaimy WK, Luczak S, Myers M, Strong D: Does e-cigarette use predict cigarette escalation? A longitudinal study of young adult non-daily smokers. *Prev Med* 2017, 100:279-284.	Wrong outcome
Doran N, Correa JB, Myers MG, Tully L: Associations Between Self-Reported and Biological Measures of Nicotine Consumption Among Young Adult Nondaily Cigarette Smokers. *Am J Addict* 2020.	Wrong outcome
D'Ruiz CD, Graff DW, Robinson E: Reductions in biomarkers of exposure, impacts on smoking urge and assessment of product use and tolerability in adult smokers following partial or complete substitution of cigarettes with electronic cigarettes. *BMC public health* 2016, 16:543.	Wrong outcome
D'Ruiz CD, O'Connell G, Graff DW, Yan XS: Measurement of cardiovascular and pulmonary function endpoints and other physiological effects following partial or complete substitution of cigarettes with electronic cigarettes in adult smokers. *Regulatory toxicology and pharmacology : RTP* 2017, 87:36-53.	Wrong outcome
Dunbar MS, Tucker JS, Ewing BA, Pedersen ER, Miles JN, Shih RA, D'Amico EJ: Frequency of E-cigarette Use, Health Status, and Risk and Protective Health Behaviors in Adolescents. *J Addict Med* 2017, 11(1):55-62.	Wrong outcome
Farsalinos KE, Romagna G, Tsiapras D, Kyrzopoulos S, Voudris V: Characteristics, Perceived Side Effects and Benefits of Electronic Cigarette Use: A Worldwide Survey of More than 19,000 Consumers. *International Journal of Environmental Research and Public Health* 2014, 11:18.	Comparison between EC and CC not possible
González-Roz A, MacKillop J: No evidence of differences in smoking levels, nicotine dependence, carbon monoxide or motivational indices between cigarette smokers and cigarette + e-cigarette dual users in two samples. *Addict Behav* 2021, 112:106543.	Wrong outcome
Jackson M, Singh KP, Lamb T, McIntosh S, Rahman I: Flavor Preference and Systemic Immunoglobulin Responses in E-Cigarette Users and Waterpipe and Tobacco Smokers: A Pilot Study. *Int J Environ Res Public Health* 2020, 17(2).	Not EC-CC dual use
Jacob P, St. Helen G, Yu L, Nardone N, Havel C, Cheung P, Benowitz NL: Biomarkers of Exposure for Dual Use of Electronic Cigarettes and Combustible Cigarettes: Nicotelline, NNAL, and Total Nicotine Equivalents. *Nicotine and Tobacco Research* 2020, 22(7):1107-1113.	Wrong outcome
Jain RB: Re-visiting serum cotinine concentrations among various types of smokers including cigarette only smokers: some new, previously unreported results. *Environ Sci Pollut Res Int* 2020.	Wrong outcome

Karasneh R, Al-Azzam S, Nusair M, Hawamdeh S: Perceptions, symptoms, and practices of electronic cigarette users: Descriptive analysis and validation of Arabic short form vaping consequences questionnaire. *PLoS One* 2021, 16(1):e0245443.	Comparison between EC and CC not possible
Kim J PhD MPH, Lee S: Daily Cigarette Consumption and Urine Cotinine Level between Dual Users of Electronic and Conventional Cigarettes, and Cigarette-Only Users. *J Psychoactive Drugs* 2020, 52(1):20-26.	Wrong outcome
Lechasseur A, Huppé C, Talbot M, Routhier J, Aubin S, Beaulieu M, Duchaine C, Marsolais D, Morissette M: Exposure to nicotine-free and flavor-free e-cigarette vapors modifies the pulmonary response to tobacco cigarette smoke in female mice. *American journal of physiology Cell physiology* 2020, 319:717-727.	Animal study
Lechner WV, Janssen T, Kahler CW, Audrain-McGovern J, Leventhal AM: Bi-directional associations of electronic and combustible cigarette use onset patterns with depressive symptoms in adolescents. *Prev Med* 2017, 96:73-78.	Wrong outcome
Lehmann K, Kuhn S, Reimer J: Electronic Cigarettes in Germany: Patterns of Use and Perceived Health Improvement. *Eur Addict Res* 2017, 23(3):136-147.	Comparison between EC and CC not possible
Majeed B, Linder D, Eissenberg T, Tarasenko Y, Smith D, Ashley D: Cluster analysis of urinary tobacco biomarkers among U.S. adults: Population Assessment of Tobacco and Health (PATH) biomarker study (2013-2014). *Preventive Medicine* 2020, 140 (no pagination).	Comparison between EC and CC not possible
Makena P, Liu G, Chen P, Yates CR, Prasad GL: Urinary Leukotriene E(4) and 2,3-Dinor Thromboxane B(2) Are Biomarkers of Potential Harm in Short-Term Tobacco Switching Studies. *Cancer Epidemiol Biomarkers Prev* 2019, 28(12):2095-2105.	Not EC-CC dual use
Makri OE, Pallikari A, Kagkelaris K, Mastronikolis SN, Karanasios G, Symeonidis C, Plotas P, Georgakopoulos CD: The Acute Effects of Electronic Cigarette Vaping and Tobacco Cigarette Smoking on Choroidal Thickness in Young, Healthy, Habitual, Dual Smokers. *Toxics* 2020, 8(4).	Comparison between EC and CC not possible
Marsden DG, Loukas A, Chen B, Perry CL, Wilkinson AV: Associations between frequency of cigarette and alternative tobacco product use and depressive symptoms: A longitudinal study of young adults. *Addict Behav* 2019, 99:106078.	Wrong outcome
Mohamed MHN, Rahman A, Jamshed S, Mahmood S: Effectiveness and safety of electronic cigarettes among sole and dual user vapers in Kuantan and Pekan, Malaysia: a six-month observational study. *BMC Public Health* 2018, 18(1):1028.	Comparison between EC and CC not possible
Nollen NL, Mayo MS, Clark L, Cox LS, Khariwala SS, Pulvers K, Benowitz NL, Ahluwalia JS: Tobacco toxicant exposure in cigarette smokers who use or do not use other tobacco products. *Drug Alcohol Depend* 2017, 179:330-336.	Not EC-CC dual use
O'Connell G, Graff DW, D'Ruiz C: Reductions in biomarkers of exposure (BoE) to harmful or potentially harmful constituents (HPHCs) following partial or complete substitution of cigarettes with electronic cigarettes in adult smokers. *Toxicology Mechanisms and Methods* 2016, 26(20):453-464.	Not real-world study
Park MB, Choi JK: Differences between the effects of conventional cigarettes, e-cigarettes and dual product use on urine cotinine levels. *Tob Induc Dis* 2019, 17:12.	Wrong outcome
Polosa R, Morjaria J, Caponnetto P, Caruso M, Strano S, Battaglia E, Russo C: Effect of smoking abstinence and reduction in asthmatic smokers switching to electronic cigarettes: evidence for harm reversal. *International journal of environmental research and public health* 2014, 11(5):4965-4977.	Comparison between EC and CC not possible
Polosa R, Morjaria JB, Caponnetto P, Prosperini U, Russo C, Pennisi A, Bruno CM: Evidence for harm reduction in COPD smokers who switch to electronic cigarettes. *Respiratory research* 2016, 17(1):166.	Comparison between EC and CC not possible
Polosa R, Morjaria JB, Prosperini U, Russo C, Pennisi A, Puleo R, Caruso M, Caponnetto P: Health effects in COPD smokers who switch to electronic cigarettes: a retrospective-prospective 3-year follow-up. *Int J Chron Obstruct Pulmon Dis* 2018, 13:2533-2542.	Comparison between EC and CC not possible
Pulvers K, Emami AS, Nollen NL, Romero DR, Strong DR, Benowitz NL, Ahluwalia JS: Tobacco Consumption and Toxicant Exposure of Cigarette Smokers Using Electronic Cigarettes. *Nicotine Tob Res* 2018, 20(2):206-214.	Comparison between EC and CC not possible
Rubinstein ML, Delucchi K, Benowitz NL, Ramo DE: Adolescent Exposure to Toxic Volatile Organic Chemicals From E-Cigarettes. *Pediatrics* 2018, 141(4).	Comparison between EC and CC not possible
Singh KP, Maremanda KP, Li D, Rahman I: Exosomal microRNAs are novel circulating biomarkers in cigarette, waterpipe smokers, E-cigarette users and dual smokers. *BMC Med Genomics* 2020, 13(1):128.	Not EC-CC dual use

Smith DM, Christensen C, van Bemmel D, Borek N, Ambrose B, Erives G, Niaura R, Edwards KC, Stanton CA, Blount BC *et al*: Exposure to Nicotine and Toxicants Among Dual Users of Tobacco Cigarettes and E-Cigarettes: Population Assessment of Tobacco and Health (PATH) Study, 2013-2014. *Nicotine Tob Res* 2021.	Comparison between EC and CC not possible
So CJ, Meers JM, Alfano CA, Garey L, Zvolensky MJ: Main and Interactive Effects of Nicotine Product Type on Sleep Health Among Dual Combustible and E-Cigarette Users. *Am J Addict* 2020.	Comparison between EC and CC not possible
Soule EK, Bode KM, Desrosiers AC, Guy M, Breland A, Fagan P: User-Perceived Negative Respiratory Symptoms Associated with Electronic Cigarette Use. *Nicotine & Tobacco Research* 2020, 22:S45-S53.	Comparison between EC and CC not possible
St Helen G, Liakoni E, Nardone N, Addo N, Jacob P, 3rd, Benowitz NL: Comparison of Systemic Exposure to Toxic and/or Carcinogenic Volatile Organic Compounds (VOC) during Vaping, Smoking, and Abstention. *Cancer Prev Res (Phila)* 2020, 13(2):153-162.	Comparison between EC and CC not possible
St Helen G, Nardone N, Addo N, Dempsey D, Havel C, Jacob P, 3rd, Benowitz NL: Differences in nicotine intake and effects from electronic and combustible cigarettes among dual users. *Addiction* 2020, 115(4):757-767.	Comparison between EC and CC not possible
Veldheer S, Yingst J, Midya V, Hummer B, Lester C, Krebs N, Hrabovsky S, Wilhelm A, Liao J, Yen MS *et al*: Pulmonary and other health effects of electronic cigarette use among adult smokers participating in a randomized controlled smoking reduction trial. *Addictive behaviors* 2019, 91:95-101.	Comparison between EC and CC not possible
Vora MV, Chaffee BW: Tobacco-use patterns and self-reported oral health outcomes: A cross-sectional assessment of the Population Assessment of Tobacco and Health study, 2013-2014. *J Am Dent Assoc* 2019, 150(5):332-344.e332.	Not EC-CC dual use
Wiener RC, Bhandari R: Association of electronic cigarette use with lead, cadmium, barium, and antimony body burden: NHANES 2015-2016. *J Trace Elem Med Biol* 2020, 62:126602.	Comparison between EC and CC not possible
Wiernik E, Airagnes G, Lequy E, Gomajee R, Melchior M, Le Faou AL, Limosin F, Goldberg M, Zins M, Lemogne C: Electronic cigarette use is associated with depressive symptoms among smokers and former smokers: Cross-sectional and longitudinal findings from the Constances cohort. *Addict Behav* 2019, 90:85-91.	Wrong outcome
Wong LP, Mohd Salim SN, Alias H, Aghamohammadi N, Hoe VCW, Isahak M, Ali Mohd M: The Association Between E-Cigarette Use Behaviors and Saliva Cotinine Concentration Among Healthy E-Cigarette Users in Malaysia. *J Addict Nurs* 2020, 31(2):102-109.	Wrong outcome

Table S3. Detailed overview of all 52 included studies investigating health effects of dual use.

**First author, reference, year of publication, country**	**Conflict of interest**	**Method**	**Duration at follow-up**	**Numbers included, description of participants and use**	**Percent dual users in the study sample**	**Risk of bias**	**Major outcomes**	**Findings**<br>Adjusted analyses and odds ratio (aOR) shown (95%confidence intervals) if not mentioned otherwise	**Overall finding: Dual use outcome**
Akinkugbe A.A.<br>(1)<br>2019<br>USA	None	Population-based survey (PATH)<br><br>Cross-sectional data<br><br>High participation rates	12 months (past year)	13,650 adolescents aged 12 to 17 years<br><br>Past 30 days use<br>12,692 NU<br>221 EC<br>433 ESCC<br>196 DU	1.4% of all<br><br>66% of EC	Low risk of selection bias<br><br>Risk of recall bias<br><br>Weighted data<br><br>Adjusted for 6 confounders	Self-reported past-year diagnosis with dental problems (told by health professional)	Ever users:<br>Never use, ref =1<br>EC: 1.11 (0.79 to 1.55)<br>ESCC: 1.50 (1.18 to 1.90)<br>DU: 1.72 (1.24 to 2.38)<br><br>Current/past 30 days users:<br>Never use, ref =1<br>EC: 1.12 (0.90 to 1.38)<br>ESCC: 1.34 (1.13 to 1.58)<br>DU: 1.43 (1.22 to 1.67)	DU: higher odds of dental problems than ESCC, but SIGN. not tested
Bhatta D. N. (2)<br>2020<br>USA	None	Nationally representativ e, longitudinal cohort study (PATH)<br><br>High participation rates	2 years retrospective respiratory disease	32,320 adults<br><br>Never, former and current smokers and EC, DU<br>Current use: every day or some days<br><br>Only weighted numbers shown	78.6% of EC users at wave 1	Low risk of selection bias<br><br>Risk of recall bias<br><br>Weighted data<br><br>Adjusted for 5 confounders	Self-reported respiratory disease (chronic obstructive pulmonary<br>disease, chronic bronchitis, emphysema, or asthma)	The total adj. odds of developing respiratory disease for a current DU= 3.30 compared with a never smoker who never used EC<br><br>Ref. never smoker who never used EC<br>DU (with TP): aOR=3.04<br>DU (with CC): aOR=3.32<br>Current ESCC: aOR=2.56 (1.92, 3.41)<br>Current EC: aOR=1.29 (1.03, 1.61)	DU: higher odds of reporting of respiratory disease than ESCC but SIGN. level not tested
Cardenas V.M.<br>(3)<br>2020<br>USA	None	A state-level, population-based public health surveillance system that monitors key maternal behaviors	Prospective design: EC exposure during the 3 months before pregnancy and/or during the last 3 months of pregnancy	1594 pregnant women<br><br>User: Daily and non-daily use in last 30 days<br>372 ESCC<br>100 DU<br>18 EC only	6.6% of all<br><br>80% of EC	Low risk of selection bias<br><br>Risk of recall and social desirability-ty bias<br><br>Weighted data<br>Adjusted for 4 confounders	Risk of small-for-gestational-age (SGA)	Estimated adjusted RR for SGA<br>ESCC: 1.7 (95% confidence interval [CI]: 1.1, 2.7),<br>DU: 1.8 (95% CI: 1.0, 3.4)<br><br>Women who were DU and continued using EC but stopped smoking cigarettes had an increased risk for SGA compared with NU, 3.2 (95% CI: 1.5, 6.6).	DU: higher odds of giving birth to a small-for-gestational-age child than ESCC, but SIGN. level not tested
Carroll D.M. (4)<br>2018<br>USA	None	Clinical cross-sectional study	No follow-up	94 persons of American Indian descent<br>Mean age 38<br><br>DU definition: smoked ≥ 5 CC in the past 3 months, smoked in the past 24 hours, used an EC every day in the past 3 months,	33% of sample<br>54% of EC	High risk of selection bias<br><br>Adjusted for urine creatinine only<br><br>Measurement bias	NNAL carcinogen metabolite in urine<br><br>CO (cardiovascular toxicant)	NNAL (pg/mg)<br>EC: 6.1<br>ESCC: 261.4<br>DU: 228.0 (ESCC vs. DU: p = 0.35)<br><br>CO (ppm)<br>EC: 2.4<br>ESCC: 14.7<br>DU: 16.8 (ESCC vs. DU: p = 0.54)	DU same level of carcinogen biomarker and cardiovascular toxicant as ESCC

				used an EC in the past 24 hours, and had not used any other tobacco products in the past 3 months.<br>34 ESCC<br>31 DU<br>29 EC only					
Chen D. TH. (5)<br>2021<br>UK	None	Cross-sectional study<br><br>Data from wave 1 (May 2020) of University College London Centre for Longitudinal Studies COVID-19 study.<br>Online survey.	No follow-up	N= 13,077 adults (20-63 y/o)<br><br>Self-reported non-smoker, current or occasionally ESCC, EC or DU.<br><br>287 DU<br>1198 ESCC<br>489 EC only	2.2 % of all<br><br>37% of EC	Low risk of selection bias<br><br>Risk of recall bias and of desirability bias<br><br>Adjusted for 6 confounders<br><br>Weighted data	Self-reported experience of covid-19 symptoms, adherent of social-distancing, confirmed/suspected covid-19 diagnosis, received covid-19 test.	NU= ref.<br>Experiencing Covid-19 symptoms:<br>ESCC: aOR= 1.21 (0.87, 1.69) p= 0.26<br>EC: aOR= 1.08 (0.61, 1.89) p= 0.8<br>DU: aOR= 1.41 (0.79, 2.54) p= 0.25<br><br>Confirmed/suspected covid-19 diagnosis:<br>ESCC: aOR= 1.1 (0.89, 1.36) p= 0.66<br>EC: aOR= 1.22 (0.90, 1.65) p= 0.97<br>DU: aOR=2.15 (1.15, 4.05) p= 0.02<br><br>Received covid-19 test:<br>ESCC: aOR= 1.05 (0.75,1.48) p= 0.75<br>EC: aOR= 0.85 (0.48, 1.50) p= 0.16<br>DU: aOR= 1.97 (0.62, 6.32) p= 0.25	DU had higher odds of covid-19 symptoms and higher odds of confirmed/suspected covid-19 diagnosis than ESCC but SIGN. level not tested
Cho J. H. (6)<br>2016<br>South Korea	None	Cross-sectional analysis on data from the Korea Youth Risk Behaviors Web-based survey (2014)	Past 12 months	N= 35,904 adolescents (10th – 12th grade in high school)<br><br>Current (past 30 days), former and never users of EC and ESCC.<br><br>2,513 EC<br>31,313 Never -EC<br>26,490 never- ES<br>4,694 ESCC<br>DU?	5.6 % of all	Low risk of selection bias<br><br>Adjusted for 7 confounders<br><br>Not stated that it is weighted/not weighted?	Self-reported asthma diagnosis	Never user: (ref.)<br>FormerEC+ESCC: AOR= 1.16 (0.42, 3.19)<br>FormerEC-CurrentESCC: AOR= 0.91 (0.34, 2.40)<br>CurrentEC-FormerESCC: AOR= 0.60 (0.21, 1.71)<br>DU: AOR= 0.46 0.20, 1.07)<br><br>Du higher odds of asthma compared to ESCC-neverEC and ESCC-formerEC (05% CI). Only SIGN. in DU in unadjusted model:<br>ESCC-neverEC: ref.<br>ESCC-formerEC: OR= 0.92 (0.57, 1.51)<br>DU: OR= 1.45 (1.00, 2.11), p< 0.05<br><br>ESCC-neverEC: ref.<br>ESCC-formerEC: aOR= 0.91 (0.54, 1.54)<br>DU: aOR=1.30 (0.86, 1.96)	DU higher odds of reporting asthma than ESCC but not SIGN. in adjusted analyses
Choi D-W (7)<br>2018<br>South Korea	None	National survey<br>Cross sectional data		N= 8,809 adults<br><br>Current use: not specified<br><br>142 DU<br>1359 ESCC<br>1654 Ex-ESCC<br>5654 NU	1.6% of all (no info on EC)	Low risk of selection bias<br><br>Adjusted for 12 confounders<br><br>Took former tobacco consumption into account	Diabetes (HbA1c)	DU: β: 0.1116; SE: 0.0343 (p=0.001)<br>ESCC: β: 0.0752; SE:0.0245 (p=0.002)<br>Ex-ESCC: β: 0.0261; SE: 0.0234 (p=0.26)<br>NU: ref	DU SIGN. worse than NU<br><br>DU worse than ESCC but not SIGN.

						Weighted data			
Chung S. J. (8)<br>2020<br>South Korea	None	Cross-sectional analysis on data from the Korea youth Risk Behavior Survey (2018)	Past 12 months	N= 60,040 adolescents (13-18 y/o)<br><br>Current (past 30 days), former and never use of EC, CC and HTP (HTP not included here)<br><br>1456 EC<br>3722 ESCC<br>531 DU and never user of heated tobacco	2.7 % of all<br><br>36% of EC	Low risk of selection bias<br><br>Adjusted for 8 confounders<br><br>Weighted data	Self-reported asthma and allergic rhinitis diagnosis	DU had higher odds for current allergic rhinitis than ESCC compared to ESCC but SIGN. not tested<br>NUs: ref.<br>ESCC-only: aOR= 1.3 (1.1, 1.6), p= 0.02<br>DU: aOR= 1.6 (1.2, 2.2), p= 0.002<br><br>DU had lower odds for current asthma than ESCC in adjusted analysis but SIGN. not tested.<br>Never users: ref .<br>ESCC-only: OR= 1.6 (1.1-2.2), p=0.005<br>DU: OR= 1.2 (0.8-2.0),p=0.39	DU had higher odds for current allergic rhinitis but lower odds of current asthma than ESCC, but SIGN. not tested
Clemens M.M. (9)<br>2019<br>USA	None	A state-level, population-based public health surveillance system that monitors key maternal behaviors (Important: subsample of (3))	Prospective design: EC exposure during the 3 months before pregnancy and/or during the last 3 months of pregnancy	248 pregnant women<br><br>76 singleton livebirths from 81 women<br><br>Past 30 day use<br>27 ESCC<br>11 DU<br>38 NU	6.6% of all	Low risk of selection bias<br><br>Risk of social desirability bias<br><br>Weighted data<br><br>Adjusted for 4 confounders<br>Confirmed smoking status	Carcinogen metabolites (TSNAs, NNAL, and NNK) in hair samples<br><br>Risk of small-for-gestational-age (SGA)	DU: levels of TSNAs similar to ESCC<br><br>RR for SGA, confirmed smoking status by hair nicotine level (only 58 women)<br>NU: ref.<br>DU RR= 8.3; 1.0–69.1 (p=0.05)<br>ESCC: RR= 7.8; 1.0–59.0 (p=0.05)	DU same level of carcinogen biomarkers as ESCC
Dinkeloo E. (10)<br>2019<br>USA	None	Online survey<br>Cross sectional data	Use of product in the last 30 days	2,854 men, active duty soldiers, mean age 25 years<br><br>current DU: EC use and tobacco cigarette use within the last 30 days and smoking 100 CC in their lifetime<br><br>2298 NU<br>355 ESCC<br>63 EC<br>138 DU	4% of all<br><br>69% of EC were DU	Low risk of selection bias<br><br>Risk of social desirability bias<br><br>Adjusted for 2 confounders	Physical activity (Exposure-Specific APFT Performance): 2-mile run, push-p test and sit-up test	NU averaged the most total physical training, followed by EC, ESCC, and then DU.<br>DU had significantly (p<0.05) lower scores than ESCC and NU on all 3 fitness events (2 mile run time, push-up and sit-up performance)	DU: SIGN. worse fitness than ESCC
Fetterman J. (11)<br>2020	None	Clinical study	-	N= 467 healthy adults 21-45Y	11 % of all/sample, 59% of EC	High risk of selection bias	Cardiovascular health Augmentation index (arterial stiffness)	Many measures of vascular health did not	DU

USA		Cross-sectional data collected in one visit<br><br>Noninvasive vascular function testing		Current users<br>Dual use: lifetime usage of ≥100 CC and EC usage of at least 5 days per week<br><br>94 NU<br>285 ESCC<br>36 EC only<br>52 DU		Adjusted for 4 confounders		differ between NU, EC, DU and ESCC, including measures of large and small vessel vasodilator response<br><br>In multivariable adjusted models:<br>Augmentation index values (P=1.0)<br>DU: 134.9±4.0<br>ESCC: 129.8±1.5<br>The augmentation index was similar between ESCC, EC and DU	same arterial stiffness as ESCC
Flacco M. E. (12)<br>2019<br>Italy	Yes	Cohort study<br><br>4th follow-up of study described by Manzoli 2015	48 ± 3 months	1355 adults enrolled at baseline<br>N= 915<br><br>Current users of products at least 6 months at baseline<br>228 EC only<br>471 ESCC<br>216 DU	59% of EC	High risk of recall and selection bias<br><br>63% validated by hospital records<br><br>Adjusted for 13 confounders<br><br>Took former tobacco consumption into account	Changes in self-reported health<br><br>Possibly smoking-related disease: (PSRD)<br>(COPD, myocardial infarction and/or angina, congestive heart failure, transitory cerebrovascular ischemia or stroke, any cancer)	Multivariate analyses:<br>(OR, 95% CI) of PSRD<br>observed among baseline-users: No significant differences across groups<br>ESCC ref (0)<br>EC: aOR: 1.01 (0.52-1.98), p=0.9<br>DU: aOR: 1.57 (0.84-2.96), p=0.16<br>No significant differences across groups when restricted to non-switchers.<br><br>Self-reported health score (diff. baseline)<br>ESCC: ref (0)<br>EC: -0.34 (-0.65, -0.04), p= 0.028<br>DU: 0.45 (-0.13, 1.04), p= 0.13	DU same self-reported health and same rate of smoking related disease after 4 years as ESCC
Flacco M. E. (13)<br>2020<br>Italy	Yes	Cohort study<br><br>5th follow-up of study described by Manzoli 2015	72 ± 3 months	1355 adults enrolled at baseline<br>N= 912<br><br>Current users of products at least 6 months at baseline<br>228 EC<br>469 ESCC<br>215 DU	Baseline: 15.9 % of all<br><br>Follow-up: 5.2 %	High risk of recall and selection bias<br><br>62.8 % validated by hospital records<br><br>Analyses A adjusted for 5 confounders ; analyses B adjusted for 13 confounders<br><br>Took former tobacco consumption into account	Changes in self-reported health<br><br>Possibly smoking-related disease: (PSRD)<br>(COPD, myocardial infarction and/or angina, congestive heart failure, transitory cerebrovascular ischemia or stroke, any cancer)	Multivariate analyses A:<br>(OR, 05% CI) of PSRD:<br>Observed among baseline-users: No significant differences across groups<br>ESCC: ref.<br>EC: aOR= 1.17 (0.64, 2.13) p= 0.6<br>DU: aOR= 1.48 (0.81, 2.70) p= 0.2<br>No significant differences across groups when restricted to non-switchers.<br><br>Multivariate analyses B:<br>Self-reported health score (diff. baseline)<br>ESCC: ref.<br>EC: -0.19 (-0.42, 0.05) p= 0.12<br>DU: 0.16 (-0.08, 0.39) p= 0.19<br>No significant differences across groups when restricted to non-switchers.	DU had higher odds of possibly smoking related disease after 6 years than ESCC but not SIGN.
Gaiha S. M. (14)<br>2020<br>USA	None	National online survey<br><br>Cross-sectional data	-	N= 4,351<br><br>Adolescents and young adults 13-24 years<br><br>Current (past 30 days) use<br>2,168 NU<br>2,183 EC-ever	?	Low risk of selection bias<br><br>Recall bias<br><br>Weighted data<br><br>Adjusted for 5 confounders	Self-reported COVID-19 related symptoms, testing and diagnosis	Multivariate adjusted analyses:<br>COVID-19 diagnosis in past 30 days users of product; aOR (95CI%)<br>ESCC: 1.53 (0.29, 8.14)<br>EC: 1.91 (0.77, 4.73)<br>DU: 6.84 (2.40, 19.55)<br>NU: ref<br><br>Current COVID-19 symptoms in past 30 days users of product aOR (95CI%)<br>ESCC: 1.15 (0.58, 2.27)<br>EC: 1.43 (0.84, 2.43)	DU higher risk of COVID-19 symptoms and diagnosis than NU (and probably ESCC (SIGN. not tested)

								DU: 4.69 (3.07, 7.16)<br>NU: ref	
Goniewicz M.(15)<br>2018<br>USA	Yes	Nationally representative, longitudinal cohort study (PATH) 2013-2014<br><br>Cross-sectional analyses	-	N=5,105 adults (18+)<br><br>Current every day or some day use<br>ESCC: 2411<br>EC: 247<br>DU:792 (only 20% used EC daily)<br>NU: 1655<br>(n lower for VOC metabolite analyses)	15.5 % of all<br><br>77% of EC	Low risk of selection bias<br><br>creatinine level corrected<br><br>weighted data<br><br>Measurement bias<br><br>Adjusted for 7 confounders	50 biomarkers of toxicity (TSNAs, metals, PAHs & VOCs) in urine	DU had higher geo mean in 46/50 biomarkers.<br><br>The higher concentrations of biomarkers were statistically significant compared to ESCC in 28/50 biomarkers:<br>5/8 urinary nicotine metabolites<br>3/4 TSNAs<br>2/8 heavy metals<br>5/7 PAHs<br>13/20 VOCs<br>Mean concentrations of lead and cadmium did not differ between DU and ESCC	DU: SIGN. higher concentration of most biomarkers of toxicity/carcinogenicity than ESCC
Harlow A. (16)<br>2020<br>USA	None	Prospective preconception cohort study<br><br>Online survey (PRESTO study)	Bimonthly follow-up questionnaires until self-reported pregnancy, up to 12 months	N= 4,586 women trying to conceive 21-45 years<br><br>3427 completed, 1115 lost to f-up<br><br>Current and use Baseline: ever use, follow-up: use in the previous 4 weeks.<br><br>NU: 3,432<br>? ESCC<br>Former EC:609<br>Current EC:172<br>DU: 34	0.7% of all<br>17% of EC	Low risk of selection bias<br><br>High risk of social desirability bias<br><br>Adjusted for 12 confounders<br><br>Took former tobacco consumption into account	Fecundability (menstrual cycle and achieved pregnancy)	Adjusted fecundability ratio (FR 95%CI)) compared to noncurrent users (never and former EC and/or ESCC)<br>EC: 0.91 (0.70, 1.18)<br>ESCC: 1.01 (0.85, 1.20)<br>DU: 0.83 (0.54, 1.29) (only 25 pregnancies, low power)	DU lower fecundability ratio than ESCC but not SIGN. (low power)
Hedman L. (17)<br>2018<br>Sweden	Yes	Cross-sectional analyses on data from two population-based surveys Obstructive Lung Disease in Northern Sweden (OLIN) and West Sweden Asthma Study (WSAS)<br><br>same validated questionnaire	Past year	N= 30,272 adults<br><br>Adults age 20-75<br><br>Current use:<br>ESCC: Do you smoke?<br>EC daily or occasional use<br>EC: 529<br>ESCC: 3694<br>FESCC: 7305<br>DU: 350	1.1 % of all<br><br>67% of EC	Low risk of selection bias<br><br>Recall bias<br><br>Adjusted for 4 confounders	Self-reported respiratory symptoms: long-standing cough (past year), sputum production, chronic productive cough (3 months – 2 years), any wheeze (12 months), recurrent wheeze	All symptoms were most common in DU (p<0.01).<br><br>Adjusted analyses, aOR (95%CI) for having any respiratory symptom:<br>NU: ref<br>DU: 4.03; (3.23-5.02)<br>ESCC: 2.55; (2.36-2.77)<br>FESCC: 1.27; (1.19-1.36)	DU had SIGN. more self-reported respiratory symptoms than NU<br><br>Worse than ESCC (SIGN. not tested)

Jain R. (18)<br>2019<br>USA	None	Cross-sectional analysis on data from the population-based survey NHANES (2013-2016)	-	1139 US residents aged ≥12 years<br><br>Current use of products during the last 5 days<br>ESCC: 891<br>EC only: 52<br>DU: 46<br>TP: 105	4 % of all<br><br>47% of EC	Low risk of selection bias<br><br>Weighted analyses<br><br>Adjusted analyzes, 5 confounders<br><br>Measurement bias	Levels of metals in blood (cadmium, lead and mercury)	DU and EC did not have lower levels of blood cadmium, lead, and mercury than ESCC or TP.<br>Adjusted geometric means (AGM)of cadmium:<br>DU: 0.64 (0.44-0.93)<br>ESCC: 0.78 (0.72-0.84)<br>EC: 0.7 (0.49-1.01)<br>Adjusted geometric means (AGM)of lead:<br>DU: 1 (0.78-1.29)<br>ESCC: 1.19 (1.11-1.28)<br>EC: 1.17 (0.9-1.52)<br>Adjusted geometric means (AGM)of mercury:<br>DU: 0.66 (0.45-0.97)<br>ESCC: 0.68 (0.61-0.76)<br>EC: 0.83 (0.48-1.43)	DU same levels of metals in blood as ESCC and EC
Keith R. (19)<br>2020<br>USA	None	Cross-sectional analysis on data from a cohort CITU (2014-2016)	-	371 healthy adults mean age 32 years<br><br>Current (past 30 days) use<br>NU: 87<br>EC only: 17<br>ESCC: 237<br>DU: 30	8 % of all<br>64% of EC	Low risk of selection bias<br><br>Measurement bias<br><br>Adjusted for 3 confounders	Toxicity<br><br>Volatile organic compound (VOC) metabolites in urine	ESCC and DU had SIGN. elevated levels of all VOC metabolites except MU, BPMA, and BMA<br><br>DU and ESCC had similar levels of all VOC metabolites except PGA,<br>PHEMA, and 3MHA + 4MHA, which were all significantly higher in<br>ESCC than DU<br><br>DU: lower levels of metabolites of styrene and xylene than ESCC	DU and ESCC had similar levels of most VOC metabolites
Kim C. (20)<br>2020<br>South Korea	None	Cross-sectional analysis on data from the population-based survey KNHANES (2013-2017)<br><br>Professional physiological measures and a survey	-	N= 7,505 adult men (19+)<br>Mean age 37 years<br><br>DU: 100 CC in lifetime; EC use in the past month<br>DU: 337<br>ESCC: 4,079<br>EC only: 62<br>NU: 3,027	5.1 % of all<br>84% of EC<br><br>DU: SIGN. higher poly-use of new tobacco products	Low risk of selection bias<br><br>Recall bias<br><br>Adjusted for 14 confounders<br><br>Measurement bias<br><br>Weighted data	Cardiovascular risk factors<br><br>Risk factors:<br>Elevated waist circumference (WC), blood pressure (BP), triglycerides (T) and fasting glucose (FG), reduced HDL-cholesterol (HDL-C) and diagnosis of metabolic syndrome (MetS)	Fully adjusted model:<br>DU SIGN. higher association compared to ESCC and NU. aPOR (95%CI)<br>Elevated WC<br>DU vs. ESCC: 1.96 (1.19, 3.23), p= 0.008<br>DU vs. NU: 2.26 (1.31, 3.91), p=0.003<br><br>Elevated triglycerides<br>DU vs. ESCC: 1.44 (0.99, 2.10), p= 0.058<br>DU vs. NU: 2.81 (1.90, 4.14), p<0.001<br><br>Reduced HDL-cholesterol<br>DU vs. ESCC: 1.9 (1.31, 2.76), p= 0.001<br>DU vs. NU: 2.48 (1.66, 3.71), p<0.001<br><br>Diagnosis of MetS<br>DU vs. ESCC: 1.57 (1.03, 2.40), p= 0.038<br>DU vs. NU: 2.79 (1.72, 4.53), p< 0.001<br><br>Elevated BP:<br>DU vs. ESCC: 0.68 (0.47–0.98), p=0.037<br>DU vs. NU: 0.62 (0.41–0.94), p=0.023<br><br>Elevated fasting glucose<br>DU vs. ESCC: 1.19 (0.78–1.82), p=0.425<br>DU vs. NU: 1.38 (0.89–2.16), p=0.153	DU SIGN. worse cardiovascular risk factors than ESCC<br><br>Except for fasting glucose

Kim T. (21)<br>2020<br>South Korea	None	Cross-sectional analysis on data from the 6th Korea National Health and Nutrition Examination Survey (2013-2015)	No follow-up	N= 14,738 adults (≥19 y/o)<br><br>DU: 100 cigarettes and smoke currently + EC in the past month<br><br>325 EC<br>271 DU	1.8 % of all<br>85 % of EC	Low risk of selection bias<br><br>Recall bias<br><br>Adjusted for 6 confounders<br><br>Measurement Bias<br><br>Weighted data	Metabolic syndrome, abdominal obesity, high triglyceride, high fasting glucose, low HDL-cholesterol, High blood pressure	DU significant higher association with abdominal obesity compared to ES and ESCC_everEC (aOR 95% CI), p< 0.001:<br>ESCC: ref.<br>ESCC_everEC: OR= 1.28 (1.04, 1.58)<br>DU: 1.71 (1.25, 2.34)<br><br>No significant differences between groups in other outcomes.	DU: SIGN. higher odds of abdominal obesity than ESCC<br>Other outcomes: no SIGN. difference but tendency to higher odds in DU (except blood pressure)
Kim T. (22)<br>2021<br>South Korea	None	Cross-sectional analysis on data from the nationally representative population-based survey KNHANES (2016-2017)<br><br>Professional physiological measures (urine and blood sample) and a survey		N= 10,692, adults (>19 y/o).<br><br>DU: 100 cigarettes and smoke currently + EC in the past month<br><br>9,905 NU<br>178 EC users total<br>173 DU	97.4 % of EC	Low risk of selection bias<br><br>Recall bias<br><br>Selection bias<br><br>Adjusted for 10 confounders<br><br>Measurement bias<br><br>Weighted data	Levels of serum uric acid and hyperuricemia	DU SIGN. higher prevalence of hyperuricemia among all, men, and women who smoke (p< 0.001)<br>ESCC: 14.2 % (0.9)<br>ESCC_ECever: 19.2 % (2.1)<br>DU: 26.6 % (3.6)<br><br>Fully adjusted model:<br>DU SIGN. higher association between EC use and serum uric acid levels among all, men, and women who smoke (N2,361) All (p= 0.001)<br>ESCC: 5.29 (5.18, 5.39)<br>ESCC_ECever: 5.44 (5.29, 5.59)<br>DU: 5.62 (5.41, 5.83)<br><br>DU SIGN. higher association between EC use and hyperuricemia among all and men who smoke.<br>All (p= 0.006), aOR (95%CI)<br>ESCC: (ref.)<br>ESCC_ECever: 1.17 (0.83, 1.65)<br>DU: 1.96 (1.29, 2.99)	DU SIGN. higher levels of uric acid and risk of hyperuricemia than ESCC
Leavens E. (23)<br>2020<br>USA	None	Cross-sectional analysis on data from The Minnesota Homeless Study 2015 and 2018<br><br>Interview-survey. Participants received a $10 gift card.	-	Homeless adults in Minnesota<br>N(2015)= 3627<br>N(2018)= 4148<br><br>Current (past 30 days) use<br>In 2018:<br>DU: 539<br>EC: 607<br>ESCC: 2482	13 % of all<br><br>89% of EC	High risk of selection bias<br><br>Recall bias<br><br>Adjusted for 2 confounders	Self-reported chronic health conditions	DU SIGN. higher rates of asthma and cancer compared to NU and ESCC<br><br>(Percent and 95%CI)<br>Asthma<br>DU: 28.0 % (24.2, 31.8)<br>ESCC: 21.0 % (19.4, 22.6)<br>NU: 14.7 % (12.7, 16.8)<br><br>Diff in rates:<br>DU vs. ESCC: -0.07 % (-0.11, -0.3), p< 0.01<br>DU vs. NU: -0.13 % (-0.18, -0.9), p< 0.001<br><br>Cancer<br>DU: 5.2 % (3.0, 7.5)<br>ESCC: 2.7 % (2.0, 3.3)<br>NU: 1.6 % (0.8, 2.3)<br><br>Diff in rates:<br>DU vs. ESCC: -0.02 % (-0.05, 0.00), p< 0.05<br>DU vs. NU: -0.04 % (-0.06, -0.01), p< 0.001	DU SIGN. higher rates of asthma and cancer compared to ESCC

Lee A. (24)<br>2019<br>South Korea	None	Cross-sectional data from the Korea Youth Risk Behavior Survey (2018)	-	N= 58,336 adolescents (12-18 y/o)<br><br>Ever use of EC, CC and HTP (HTP not included here)<br>8129 ESCC<br>4144 EC	57.1 % of EC users	Low risk of selection bias<br><br>Recall bias<br><br>Adjusted for 6 confounders<br>Weighted data	Self-reported asthma, allergic rhinitis and atopic dermatitis	ESCC significant higher odds of asthma than DU (not signf.) compared to never users (95% CI).<br>ESCC: aOR= 1.30 (1.08, 1.56)<br>DU: aOR= 1.14 (0.84, 1.54)<br><br>DU higher odds of allergic rhinitis than ESCC compared to never users. Not significant.<br>ESCC: aOR= 1.02 (0.94, 1.10)<br>DU: aOR= 1.10 (0.99, 1.21)<br><br>DI significant higher odds of atopic dermatitis than ESCC compared to never users.<br>ESCC: aOR= 1.20 (1.07, 1.33)<br>DU: aOR= 1.24 (1.06, 1.46)	DU har lower odds of asthma than ESCC, but comparable odds of allergic rhinitis and atopic dermatitis. SIGN. not tested
Li D. (25)<br>2020<br>USA	Yes	Cross-sectional analysis on data from population-based PATH study wave 2 (2014-2015)	-	N= 28,171 adults (18+)<br><br>smoked at least 100 cigarettes in their lifetime, and currently smoke every day or some days<br><br>EC: 641 only<br>ESCC: 8525<br>DU: 1106<br>NU: 17899	3.9 % of all<br><br>63% of EC	Low risk of selection bias<br><br>Risk of recall bias<br><br>Confounding taken into account (11 variables)<br><br>Weighted data	Respiratory symptoms<br><br>Self-perceived physical and mental health	DU reported higher percentage of mental health compared to ESCC, SIGN. not tested<br>DU: 7.46 % (6.01, 9.26)<br>ESCC: 5.01 (4.54 to 5.53)<br>No SIGN. differences were found between DU and ESCC in risk of wheezing and related respiratory symptoms (aOR=1.06, 95% CI: 0.91 to 1.24).	DU same odds of respiratory symptoms as ESCC
Mainous A. (26)<br>2020<br>USA	None	Cross-sectional analysis on data from nationally representative survey, NHANES 2015-2016	-	Adults 20+ years<br>Unweighted N= 4,659.<br><br>Weighted N= 206,172,949<br><br>DU: 100 CC in lifetime; EC in the past month<br>NS, EC, ESCC or DU.	Not specified	Low risk of selection bias<br><br>Weighted data<br><br>Adjusted for 7 confounders<br><br>Measurement bias	Biomarker of inflammation and predictor of cardiovascular disease (CRP)	DU had the highest prevalence of elevated hs-CRP (>3 mg/l) (45,6 %)<br><br>DU had SIGN. higher odds of elevated hs-CRP compared with ESCC in adjusted analyses<br>DU: aOR= 2.13 (1.35, 3.37)<br>ESCC: aOR= 1.38 (1.07, 1.78)<br><br>DU had the highest level of serum cotinine.	DU SIGN. higher probability of elevated CRP than ESCC
Manzoli L. (27)<br>2015<br>Italy	None stated, the first 2 years of study were unfunded<br><br>Please see study by Flacco (12)	Cohort study<br><br>Questionnaire at baseline, and follow-up<br><br>Recruited by general practitioners and e-	12 months	1355 adults at baseline<br>30-75 years old<br><br>959 with 1-year data<br><br>Current (past 6 months) use<br>236 EC<br>491 ESCC	17.1 % of all<br>50% of EC	High risk of selection bias<br><br>Risk of recall bias<br><br>Adjusted for 13 confounders<br><br>Took former tobacco	Self-reported health	Self-reported health at 1 year, mean (SD):<br>EC: 8.0 (1.3)<br>ESCC: 7.8 (1.3)<br>DU: 7.7 (1.2)<br><br>Difference in the self-reported health score from 12 months to baseline: aOR (95%CI)<br>ESCC: ref =0<br>DU: 0.14 (-0.13; 0.40)<br>EC: 0.31 (0.04; 0.59)	DU same self-reported health as ESCC

		cigarette shops, via internet and social networks		232 DU		consumption into account		DU had similar quitting rate, no difference in self-related health, non-significant reduction in cigarettes smoked pr. day.	
Manzoli L. (28)<br>Same study as (12) (13) and (27)<br>2017<br>Italy	None stated, the first 2 years of study were unfunded<br><br>Please see study by Flacco (12)	Cohort study<br><br>Questionnaire at baseline and 12 and 24 months follow-up<br><br>CO validation in 25 % of study population	24 months	1355 adults at baseline<br>30-75 years old<br><br>959 with 2-year data<br><br>Current (past 6 months) use<br>229 EC only<br>480 ESCC<br>223 DU	16.5 % of total population<br>49% of EC	High risk of selection bias<br><br>Risk of recall bias<br><br>Adjusted for 13 confounders<br><br>Took former tobacco consumption into account	Self-reported health	Self-rated health and adverse events were similar between all groups.<br>Improvement in self-reported health in baseline DU and ESCC who switched to EC (p< 0.05).<br><br>DU at baseline remained SIGN. more likely to report a serious adverse event than ESCC (OR 2.40; 95% CI 1.09 to 5.26; p=0.029).<br>DU experienced highest % in adverse effects (6.3 %, p< 0.005)<br><br>No significant difference in the proportion of participants that achieved complete abstinence and reduced tobacco consumption by 50 % or more, or by ≥5 cigarettes pr. day in all groups (p> 0.05).<br><br>DU (52,5 %) and ESCC (13,7 %) reduced cigarette consumption with ≥50 % (p< 0.001)	DU: same self-rated health and adverse events as ESCC
McDonell BP (29)<br>2020<br>Ireland	None	Pregnancy Risk Assessment Monitoring Prospective cohort study	From first hospital visit till birth	322 pregnant women<br><br>current self-reported smoking and vaping status<br>218 EC only<br>195 DU<br>99 ESCC	47% of EC	Low risk of selection bias<br><br>Social desirability bias<br><br>Adjusted for 4 confounders	Infant birthweight, gestation at delivery,<br>incidence of low birthweight	Mean gestation at delivery and mean Apgar scores were similar in all three groups<br><br>Mean birthweight and birth centile of EC was similar to that of NU and SIGN. greater than that of ESCC<br>Birth weigth EC: 3470 ±555 g (ref)<br>DU: 3140±628 g, p< 0.001<br>ESCC: 3166± 502 g, p< 0.001<br>NU: 3471 ±504 g, p=0.97<br><br>Neonatal intensive care unit admission:<br>EC: 15 (6.9%) ref<br>DU: 15 (7.6%)<br>ESCC: 6 (6%)<br>NU: 5 (4.6%)<br><br>Incidence of birthweight <10th centile<br>EC: 24 (11%) ref<br>DU: 60 (30.7%)<br>ESCC: 28 (28%)<br>NU: 14 (12.9%)	Mean birthweight and birth centile of EC was similar to that of NU and SIGN. greater than that of ESCC<br>DU: same birthweight as ESCC<br>DU: more frequent admission to neonatal intensive care unit and higher incidence of birthweight <10th centile than ESCC but SIGN. level not tested
McRobbie H. (30)<br>2015<br>UK	Yes	Experimental prospective study<br><br>Smokers should use EC ad libitum	4 weeks	N=44 smokers wanting to stop, all ESCC at baseline<br><br>Current use<br>33 at follow-up:	51.5 % of EC in sample	High risk of selection bias<br><br>Measurement bias	Measured cardiovascular toxicity (CO), and toxicity (urinary 3-HPMA, a major metabolite of acrolein)	Changes in CO from baseline to 4 weeks:<br>DU: -52 % (p= 0.001)<br>EC: -80 % (p< 0.001)<br><br>Changes in 3-HPMA from baseline to 4 weeks<br>DU: -60 % (p< 0.001)<br>EC: -79 % (p< 0.001)	DU had SIGN. reductions in toxicity after switching from ESCC in experimental setting

				16 EC<br>17 DU<br><br>DU: Reduction from 21 CPD to very little (seven had smoked one to five cigarettes in the last week and 10 had smoked more than 5)				Changes in cotinine<br>DU: -44 % (p= 0.010)<br>EC: -17 % (p= 0.486)	
Merianos A. (31)<br><br>2021<br><br>USA	None	Secondary data analysis of the school based 2017 Youth Risk Behavior Survey	-	11,296 high school students<br><br>Use in the past 30 days<br><br>566 EC only<br>157 ESCC<br>235 DU	2% of all<br><br>29% of EC	Low risk of selection bias<br><br>Adjusted for 8 covariates<br><br>Weighted data	Self-reported hours of sleep on an average school night Insufficient sleep was defined as <8 h/night and <7 h/night	Insufficient sleep <8 h/night<br>ESCC: 1 ref<br>EC: 3.20 aOR (95%CI = 1.65–6.22)<br>DU: 3.26 aOR (95%CI = 1.51–7.03) to<br><br>Insufficient sleep <7 h/night<br>ESCC: 1 ref<br>DU; 1.89 times more likely (95%CI = 1.01–3.51)	DU were SIGN. more likely to report insufficient sleep compared with ESCC
Miller C. R. (32)<br><br>2021<br><br>USA	Yes	Cross-sectional analysis on data from PATHS wave 3 (2015-2016)	-	N= 19,147 adults (18-54 y/o)<br><br>Dual use: had smoked at least 100 CC in a lifetime, and currently smoked and vaped every day or some days<br>1100 EC all<br>5654 ESCC<br>581 DU	3 % of all<br>53% of EC	Low risk of selection bias<br><br>Recall bias<br><br>Adjusted for 12 confounders<br><br>Weighted data	Self-reported diagnosis of hypertension in the last 12 months	DU had highest prevalence of self-reported hypertension in weighted analysis:<br>NS: 14.6%, EC-former ESCC: 22.5%, EC: 7.6%, former ESCC: 20.9%, ESCC: 22.4% and DU: 23.8%<br><br>DU had significant higher odds for hypertension than former ESCC and ESCC compared to NS (95 % CI):<br>Former ESCC: aOR= 1.28 (1.05, 1.57)<br>ESCC: aOR= 1.36 (1.15, 1.62)<br>DU: 1.77 (1.32, 2.39)<br><br>DU had the highest odds for hypertension than any other group compared to ESCC and former ESCC, but results were not significant.	DU had higher odds for hypertension than ESCC, but SIGN. difference not reached (0.99 for lower 95%CI)
Orimoloye O. (33)<br>2019<br>USA	None	Cross-sectional analysis on data from population-based survey NHANES (2013-2014 & 2015-2016)	-	N= 3415 adults (18+)<br><br>EC: last 5 days use<br>ESCC: smoked within the last year<br>2636 NU<br>711 ESCC<br>30 EC<br>38 DU	1 % of all<br>56% of EC	Low risk of selection bias<br><br>Analyses adjusted for 6 confounders<br><br>Measurement bias<br><br>Weighted data	Insulin resistance (measured by HOMA-IR and GTT levels)	No statistically significant association between insulin resistance and product use	DU same risk of insulin resistance as ESCC, EC and NU
Osei A. (34)<br>2019<br>USA	None	Cross-sectional analysis on data from the nationally representativ	-	N=449,092 adults (18+)<br><br>Current use<br>Dual use: had smoked at least 100 CC in	2.9 % of all<br><br>49% of EC	Low risk of selection bias<br><br>Risk of recall bias<br><br>Weighted data	Self-reported cardiovascular disease (CVD); told by doctor (Has a doctor, nurse, or other health professional ever told you that you had a stroke, myocardial infarction or coronary heart disease?)	DU had higher odds of CVD compared with ESCC and EC and the odds increased with daily use<br><br>Risk of cardiovascular disease, adjusted OR (95%)<br>ESCC: ref<br>DU (all): 1.36 (1.18, 1.56)<br>DU_occasional: 1.30 (1.12, 1.52)	DU had SIGN. higher risk of CVD than ESCC

		e, cross-sectional telephone survey BRFSS 2016-2017		a lifetime, and currently smoked and vaped every day or some days<br><br>390,303 NU<br>15,863 EC<br>58,789 ESCC<br>12,908 DU		Adjusted for 9 confounders		DU_daily: 1.59 (1.20, 2.08)<br><br>Risk of premature cardiovascular disease, adjusted OR (95%)<br>ESCC: ref<br>DU (all): 1.45 (1.20, 1.74)<br>DU_occasional: 1.36 (1.11, 1.66)<br>DU_daily: 1.84 (1.32-2.56)	
Osei A. (35)<br>2020<br>USA	Yes	Cross-sectional analysis on data from the nationally representative, cross-sectional telephone survey BRFSS 2016-2017	-	N= 705,159 adults (18+)<br><br>Current use<br>25,175 EC<br>64,792 ESCC<br>432,462 NU<br>14,036 DU	2.0 % of all<br><br>36% of EC	Low risk of selection bias<br><br>Risk of recall bias<br><br>Weighted data<br><br>Adjusted for 5 confounders	Self-reported COPD, told by doctor<br>(Has a doctor, nurse, or other health professional ever told you that you have chronic obstructive pulmonary disease or COPD, emphysema, or chronic bronchitis?)	DU had SIGN. higher odds of COPD compared with ESCC and EC<br><br>Risk of COPD, adjusted OR (95%)<br>ESCC: ref<br>DU: 1.66 (1.50, 1.84)<br>DU_occasional: 1.67 (1.50, 1.86)<br>DU_daily: 1.64 (1.34, 2.00)<br><br>DU compared to NU had the highest odds for COPD compared to any other group<br>DU: OR= 6.89 (6.29, 7.55)	DU had SIGN. higher odds of COPD than ESCC
Parekh T. (36)<br>2019<br>USA	None	Cross-sectional analysis on data from the nationally representative, cross-sectional telephone survey BRFSS 2016-2017	-	N= 161.529 young adults (18-44)<br><br>Current use<br>133,077 NU<br>13,318 ESCC<br>7,641 EC<br>7,493 DU	5 % of all<br><br>50% of EC	Low risk of selection bias<br><br>Risk of recall bias<br><br>Weighted data<br><br>Adjusted for 13 confounders	Self-reported stroke	DU had higher risk of stroke than any other group<br><br>Risk of stroke, adjusted OR (95%)<br>NU ref.<br>EC: 0.69 (0.3-1.4) (p= 0.69)<br>ESCC: 1.59 (1.1-2.2) (p< 0.01)<br>DU: 2.91 (1.6-5.3) (p<0.01)<br><br>Risk of stroke, adjusted OR (95%)<br>ESCC: ref<br>DU: 1.83 (1.1-3.2) (p< 0.05)	DU SIGN. higher risk of stroke than ESCC
Parekh T. (37)<br>2020<br>USA	None	Cross-sectional analysis on data from the nationally representative, cross-sectional telephone survey BRFSS 2016-2017	-	N=161.965 young adult women (18-44)<br><br>Current use<br>3125 DU<br>76161 NU<br>2572 EC<br>6583 ESCC (no EC use prev.)	3 % of all<br><br>55% of EC	Low risk of selection bias<br><br>Risk of recall bias<br><br>Weighted data<br><br>Adjusted for 9 confounders	Self-reported COPD and asthma<br>(Ever told that you had asthma?)<br>(Ever told you have COPD, emphysema, or chronic bronchitis?)	DU had highest odds for self-reported asthma and COPD<br><br>Risk of asthma, adjusted OR (95%)<br>NU: ref<br>EC: 1.74 (1.29, 2.35)<br>ESCC: 1.49 (1.25, 1.77)<br>DU: 2.11 (1.72, 2.59)<br><br>Risk of COPD, adjusted OR (95%)<br>NU: ref<br>EC: 1.37 (0.71, 2.63)<br>ESCC: 3.28 (2.62, 4.12)<br>DU: 5.07 (3.91, 6.56)	DU SIGN. higher risk of asthma and COPD compared with NU; higher risk than ESCC, but SIGN. not tested

Piper M (38)<br>2018<br>USA	None	Cross-sectional analysis<br>Baseline data from a 2-year cohort	-	422 adults (18+)<br><br>DU: EC use once a week for 3 months and smoked daily 3 months<br>66 ESCC<br>256 DU<br><br>Changes in DU criteria 6 months in	61 % of sample	Low risk of selection bias<br><br>Adjusted for 4 confounders , including psychiatric history<br><br>Measurement bias	Toxicity<br>NNAL and CO	CO levels (p=0.67)<br>ESCC: 16,7<br>DU: 16,3<br><br>NNAL levels (p= 0.001)<br>ESCC: 453.31<br>DU: 340.99<br>Effects were consistent when baseline demographic variables that differed between the two groups were included as covariates<br><br>DU smoked less cigarettes, were less likely to smoke within 30 minutes after waking up and had higher FTCD scores but were also more likely to have a psychiatric history.<br>Similar cotinine levels in ESCC and DU	ESCC had SIGN. higher levels of NNAL but same levels of CO as DU
Prokopowicz A.<br>(39)<br>2019<br>Poland	Yes	Cross-sectional study<br><br>Questionnaire and blood sample	-	156 young volunteer adults (19-39 years old) not occupationally exposed to Cadmium (Cd) and Lead (Pb)<br><br>DU: CC for at least 2 years and EC for at least 6 months<br>51 NU<br>48 EC<br>29 DU<br>28 ESCC	38% of EC	High risk of selection bias<br><br>Analyses adjusted for 2 confounders<br><br>Measurement bias	Harmful metals Cadmium (Cd) and Lead (Pb) in blood	Geometric mean blood Cd concentrations (adjusted for age+sex)<br>NU: 0.31 (0.26, 0.36)<br>EC: 0.44 (0.37, 0.52)<br>DU: 1.38 (1.11, 1.72), not SIGN. from ESCC<br>ESCC: 1.44 (1.16, 1.78)<br><br>Geometric mean blood Pb concentrations (adjusted for sex)<br>NU: 11.9 (10.6, 13.3)<br>EC: 14.2 (12.5, 16.0)<br>DU: 13.9 (11.9, 16.2), not SIGN. from ESCC<br>ESCC: 15.9 (13.6, 18.6)	Levels of harmful metals (cadmium and lead) not SIGN. different between DU and ESCC
Prokopowicz A.<br>(40)<br>2020<br>Poland	Yes	Cross-sectional study<br><br>Questionnaire and urine sample	-	88 young volunteer adults (19-39 years old)<br><br>DU: CC for at least 2 years and EC for at least 6 months<br>25 EC<br>25 ESCC<br>25 NU<br>13 DU	50% of EC sample	High risk of selection bias<br><br>Adjusted analyses for 3-5 confounders<br><br>Measurement bias	11 toxic metals in urine	Various tests showed no significant difference between the groups.<br><br>After controlling for age, BMI and sex, a SIGN. higher level was observed only for urinary antimony (Sb; possibly carcinogenic to humans) in DU compared to NU	DU had SIGN. higher urinary level of a toxic metal, antimony than NU
Riehm K. E. (41)<br>2019<br>USA	Yes	Cross-sectional analysis on data from a nationally representative cohort, PATH Wave 1+2.	Past year (12 months)	9.588 adolescents 12-17years old<br><br>Past 12 month use (any)<br>294 ESCC<br>474 DU<br>588 EC<br>8232 NU	5 % of all<br><br>45% of EC	Low risk of selection bias<br><br>Recall bias<br><br>Weighted data<br><br>Adjusted for many confounders	Sleep-related complaints	Statistically SIGN. association between sleep-related complaints and EC + DU in all 4 models. Final, full adjusted model (among others for lifetime-depression and life-time sleeping problems), aOR 95% CI<br>EC: 1.29 (1.05, 1.59)<br>DU: 1.57 (1.24,1.99)<br>ESCC: 1.30 (0.98,1.71)<br><br>Post hoc analysis indicates that odds for sleep-related complaints were not higher in DU than EC.	DU higher risk of sleep-related complaints than ESCC but not SIGN.<br><br>DU higher risk of sleep-related complaints than NU

Rostron B. L.<br>(42)<br>2019<br>USA	None	Cross-sectional analysis on data from a nationally representativ e cohort PATH Wave 1 (2013-2014)	-	2.710 adults<br><br>Dual use:<br>currently used CC every day and EC every day or some days<br><br>1952 ESCC<br>648 DU<br>110 ESCC+ ST (smokeless tobacco)	24 %	Low risk of selection bias<br><br>Weighted data<br><br>Adjusted for 4 confounders	Toxicity<br>Biomarkers measured in urine and blood samples	DU had higher levels of TNE2, NNAL, 1-HOP, HPMA and MHB3 compared to ESCC<br><br>ESCC+ST had higher levels of NNAL and NNN compared to ESCC	DU have higher levels of some harmful (toxic and carcinogenic) biomarkers compared to ESCC
Sanou A. Z. (43)<br>2020<br>USA	None	Register study with retrospective data collection from a cohort followed in the US Defense Medical Surveillance System which includes inpatient and outpatient medical encounter data and the annual Health Assessment	Incidence in last 9 months	802.621 adult military members<br><br>Current (past 30 days) use<br>37,915 EC<br>91,135 ESCC<br>22,010 DU<br>651,561 NU	3% of all<br><br>37% of EC	Low risk of selection bias<br><br>Risk of social desirability bias<br><br>Analyses adjusted for 4 confounders	Incident cases of acute respiratory infections (ARI)<br><br>Register data, ICD-9 and 10 codes, in- or out patients	Highest incidence rate of ARI for DU compared to ESCC, EC and NU.<br><br>Adjusted incidence rate ratios (95% CI) for ARI<br>NU: ref<br>EC: 1.02, 0.99–1.04, p= 0.123<br>ESCC: 1.01, 0.99–1.03, p=0.304<br>DU: 1.04, 1.01–1.07, p=0.021	DU had SIGN. higher risk of acute respiratory infections than NU, higher than ESCC but difference between DU and ESCC not tested
Shahab L.(44)<br>2017<br>UK	Yes	Cross-sectional study with self-selected sample	-	181 adults with long-term use of a nicotine product<br><br>Current (past 6 months) use<br>37 ESCC<br>36 ESCC-NRT<br>36 NRT<br>36 DU<br>36 EC	20% of sample	High risk of selection bias<br><br>Measurement bias<br><br>Adjusted for 3 confounders<br><br>Took former tobacco consumption into account	Carcinogen and toxin exposure<br><br>Biomarkers measured in urine and saliva samples, VOCs and TSNAs	DU higher nicotine levels than any other group<br><br>TSNA levels: Compared with ESCC there were no large differences in NNAL levels for DU<br><br>VOC level: ESCC+NRT, DU og ESCC had very similar urinary VOC metabolites levels<br><br>DU had higher VOC metabolites PMA, MU and PHEMA levels than any other group<br><br>DU: PMA (carcinogenic) SIGN. higher than in ESCC<br>Geometric mean (95% CI)<br>DU 1.43 (1.11-1.83)<br>ESCC: 0.64 (0.48-0.84)<br>ESCC+NRT: 0.44 (0.30-0.63)	DU and ESCC had similar levels of toxic and carcinogenic substances, but DU had SIGN. higher level of one carcinogenic substance, benzene than ESCC

Smith D. (45)<br>2020<br>Poland, UK and USA	Yes	Secondary analysis on data from international cross-sectional study with self-selected sample in three countries<br><br>Participants from UK, Shahab 2017, also included in this study	-	456 adults with long-term use of EC or CC use<br>US=166<br>UK=129<br>PL=161<br><br>Current (past 6 months) use<br>124 EC<br>95 DU<br>127 ESCC	US: 17%<br>UK: 28%<br>PL: 19% of sample	High risk of selection bias<br><br>Measurement bias<br><br>Adjusted for 3 confounders and for multiple comparisons	Carcinogen and toxin exposure<br><br>Biomarkers measured in urine and saliva samples, VOCs, TSNAs and minor tobacco alkaloids	DU: comparable levels of nicotine as ESCC<br><br>DU: no statistically SIGN. differences in minor tobacco alkaloids or most measured VOCs<br><br>SIGN. lower levels of acrylonitrile (CYMA) and three TSNAs in DU compared with ESCC.<br>DU in UK showed higher levels of several biomarkers of harm compared with US and Polish DU (difference in product use)<br><br>Characteristics: DU smoked fewer CPD than CS. No differences in number of puffs on e-cigarette per day between EC and DU. Nicotine intake: EC had higher levels of several nicotine metabolites compared with CS and DU.	DU and ESCC had similar levels of toxic and carcinogenic substances, but ESCC had SIGN. higher level of three TSNAs and acrylonitrile than DU
Vindhyal M. (46)<br>2020<br>USA	None	Cross-sectional study nationally representative survey<br><br>Data from NHIS (2014, 2016, 2017, 2018)	-	16.855 adults<br><br>Dual use: every or some days CC and EC<br><br>2,848 NU<br>7,291 TP<br>401 EC<br>2,240 DU<br>1,139 Former TP, now EC<br>2,936 Former TP	13% of all<br><br>40% of EC	Low risk of selection bias<br><br>Risk of recall bias<br><br>Weighted data<br><br>Adjusted for 3 confounders	Self-reported disease – told by doctor/health professional	DU had highest odds of myocardial infarction than any other group, aOR (95%CI)<br>NU: ref<br>TP: 4.52 (2.49-8.21)<br>EC: 4.09 (1.29-12.98)<br>DU: 5.44 (2.90-10.22)<br>F-TP now EC: 3.71 (1.89-7.28)<br><br>DU had highest odds of stroke than any other group, aOR (95%CI)<br>NU: ref<br>TP: 2.15 (1.38-3.35)<br>EC: 1.22 (0.36-4.18)<br>DU: 2.32 (1.44-3.74)<br>F-TP, now EC: 1.92 (1.12-3.29)<br><br>For coronary heart disease DU has almost the same odds as former ESCC (OR=2.27)	DU had higher odds of self-reported (told by doctor) myocardial infarction and stroke than EC and ESCC, but SIGN. level not tested
Wang J. B. (47)<br>2018<br>USA	Yes	Cross-sectional study using available baseline data (2013-2017) from the ongoing internet-based Health eHeart survey	Retrospective symptoms past month; cardio-pulmonary conditions "ever told by doctor"	39.747 adults<br><br>DU: 100 CC; EC in the past month<br>573 EC<br>1,693 ESCC<br>514 DU	1.3% of all<br><br>47%	Low risk of selection bias<br><br>Risk of recall bias<br><br>Adjusted for 8 confounders	Health outcomes including cardiopulmonary symptoms and conditions	DU exhibited worse median general health scores SF-12 (p= 0.002) and breathing scores (p= 0.001) in the past month compared to ESCC<br><br>No SIGN. difference in medical 19 cardiopulmonary conditions between DU and ESCC; only having a history of an arrhythmia was found to be SIGN. higher in DU (17.8%) than in ESCC (14.2%), after adjusting for covariates (p = 0.02)	DU exhibited worse median general health scores and breathing scores and had SIGN. higher prevalence of history of an arrhythmia than ESCC
Wang X. (48)<br>2020<br>USA	None	Data from Pregnancy Risk Assessment Monitoring System	3 months before pregnancy and 3 months before birth	N= 31,973 women<br><br>N(weighted)= 1,918,031<br><br>Use during the 3 months before	265 before pregnancy (3 %)<br><br>265 Late pregnancy (0,8 %)	Low risk of selection bias<br><br>Social desirability bias	Preterm birth or small-for-gestational-age (SGA)	OR* associated with preterm birth and SGA, adjusted for pre-pregnancy product use (model 2 adjusted for use of EC/ESCC before pregnancy)<br>Preterm birth<br>ESCC: aOR= 1.6 (1.2, 2.0)<br>EC: aOR= 1.2 (0.5, 2.7)<br>DU: aOR= 1.3 (0.8, 2.3)	DU had lower odds of preterm birth than ESCC, but SIGN. not tested

		(PRAMS) from 2016.<br><br>High participant rate: 83 % of all U.S. births.		their pregnancy, and during the last 3 months of pregnancy<br><br>Mothers that had singleton births in the U.S.<br><br>5029 ESCC<br>976 DU		Adjusted for 11 confounders<br><br>Weighted data		SGA<br>ESCC: aOR= 2.4 (1.8, 2.9)<br>EC: aOR= 2.4 (1.0, 5.7)<br>DU: aOR= 2.3 (1.3, 4.1)<br><br>*no p-value	DU and ESCC had same risk of small-for-gestational-age
Wills T. A. (49)<br>2019<br>USA	None	Cross-sectional analysis on 2016 Behavioral Risk Factor Surveillance Survey on Hawaii (BRFSS)	-	N= 8,087 adults (18+)<br><br>Smoked/vaped every day or some day<br><br>235 EC<br>934 ESCC	?	Low risk of selection bias<br><br>Weighted data<br><br>Adjusted for 7 confounders	Self-reported diagnosis of asthma and COPD (told by doctor/nurse)	Asthma<br>No SIGN. difference between ESCC and DU, p= 0.94<br>None: ref.<br>DU: aOR= 1.26 (1.04, 1.53), p= 0.02<br>ESCC: aOR= 1.27 (1.10, 1.47), p= 0.001<br><br>COPD<br>Absolute risk from multivariate model for COPD was highest for dual users in all age groups<br>DU significant higher odds than ESCC and EC compared to NU (95% CI):<br>NU: ref.<br>DU: aOR= 3.92 (2.82, 5.44), p< 0.0001<br>ESCC: 2.98 (2.34, 3.78), p< 0.0001<br>EC: 2.58 (1.36, 4.89), p= 0.004<br><br>Higher aOR for DU compared to ESCC and EC, but not significant:<br>DU vs. EC:1.52 (0.81, 2.87), p= 0.20<br>DU vs. ESCC: 1.32 (0.98, 1.77), p= 0.70	DU and ESCC same odds of asthma.<br>DU higher odds of COPD than ESCC but not SIGN.
Wills T. A. (50)<br>2020<br>USA	None	Cross-sectional analysis on 2017 Youth Risk Behavior Survey	Past 30 days	N= 14,765 adolescents<br><br>Ever, never and current use of EC, CC, and marijuana (marijuana not included here)<br>Past 30 days use:<br>1,659 EC<br>1,269 ESCC	?	Low risk of selection bias<br><br>Risk of recall bias<br><br>Adjusted for 5 covariates<br><br>Weighted data	Self-reported asthma diagnosis	DU SIGN. higher odds of asthma than NU (ESCC not SIGN. higher odds). Higher odds in DU than ESCC but not SIGN. p=0.10<br>Nonuser: ref.<br>EC: aOR= 1.29 (1.07, 1.55), p= 0.01<br>ESCC: aOR= 1.23 (0.92, 1.64), p= 0.17<br>DU: aOR= 1.62 (1.32, 1.99), p< 0.0001<br><br>A sensitivity analysis tested in a sample with complete data:<br>NU: ref<br>Current DU vs. ESCC: aOR 1.35 (1.03-1.76) p= 0.03). (an increment in absolute risk attributable to EC above and beyond that for smoking)	DU higher odds of asthma and past 30-day use than ESCC, but not SIGN.<br>Complete data, current users: DU had higher risk of asthma than ESCC
Xie Z. (51)<br>2020<br>USA	None	Cross-sectional using data from nationally	-	887,182 with smoking and vaping status<br><br>DU: have smoked at	1.8 % of all<br><br>64% of EC users	Low risk of selection bias<br><br>Recall bias<br><br>Weighted data	Self-reported COPD diagnosis (told by doctor)	Association between vaping and self-reported COPD diagnosis: aOR, 95% CI<br>NU: ref<br>DU: 4.39 (3.98, 4.85)<br><br>ESCC: ref	DU had SIGN. higher risk of self-reported (told by doctor) COPD than ESCC

		representativ<br>e survey<br>BRFSS survey<br>(2016 and<br>2017).		least 100 CC in<br>life, now smoke<br>every day or some<br>days, and<br>currently vaping<br>every day or some<br>days.<br><br>15,986 DU<br>115,189 ESCC<br>8876 EC only		Analyses adjusted<br>for 9 confounders		DU: 1.16 (1.05, 1.27)<br><br>Association in different age-groups:<br>DU did not show significantly higher association<br>with self-reported COPD diagnosis in 18–34 age<br>group compared to ESCC<br><br>DU: COPD diagnosis became significantly higher in<br>35–64 and over 65 age groups, with aOR = 1.15<br>(1.02 to 1.30) and aOR = 1.57 (1.22 to 2.00),<br>respectively compared to ESCC<br><br>aOR (95% CI) for COPD in 65+ years old:<br>NU: ref<br>DU: 8.38 (6.57, 10.68)<br>ESCC: 5.35 (4.87, 5.89)<br>EC: 2.48 (0.81, 7.53)<br>FESCC: 3.21 (2.98, 3.45)	
Ye D. (52)<br>2020<br>USA	None	Cross-<br>sectional,<br>pilot<br>experimental<br>study; saliva<br>sample +<br>questionnair<br>e		N=48<br><br>Smoking and<br>vaping status not<br>defined in detail<br>12 ESCC<br>12 DU<br>12 EC<br>12 NU	25 % of<br>sample	High risk of<br>selection bias<br><br>Measurement<br>bias	Systemic inflammation,<br>oxidative stress, angiogenesis<br>and tissue injury/repair in<br>saliva<br>and gingival crevicular fluid<br>(GCF)	Smoking/vaping produces<br>differential effects on oral health<br><br>PGE2: SIGN. higher in ESCC vs. NU, EC and DU.<br><br>SIGN. higher EN-RAGE (DU vs. NU), MPO (DU vs.<br>EC), Uteroglobin (DU vs. EC).<br><br>Most GCF mediators were higher for DU than<br>ESCC, but not statistically significant<br><br>DU had higher OR in all age groups compared to<br>NU, increased with increasing age. Compared with<br>ESCC significantly higher association from age-<br>group 35	DU had non-<br>SIGN. higher<br>levels of most<br>biomarkers<br>than ESCC<br><br>No SIGN.<br>difference<br>between ESCC<br>and DU in<br>biomarkers of<br>systemic<br>inflammation

▽ Conflict of interest: pharmaceutical industry
△ Conflict of interest from anonymous contributors
▲ Conflict of interest with the tobacco or e-cigarette industry
⇨ Prospective design

EC= e-cigarettes
ECU= e-cigarette users
DU= dual use/users of conventional cigarettes and e-cigarettes
ESCC= exclusive smokers/smoking of conventional cigarettes
TPU= users of combustible tobacco products (ES, cigarillos, cigars, pipe, water pipe)
NU= Non-user of conventional cigarettes and e-cigarettes
1-HOP= 1-hydroxypyrene (PAH)
3-HPMA =Urinary 3-hydroxypropyl mercapturic acid, a major metabolite of acrolein

CO= carbon monoxide HPHC = harmful and potentially harmful constituents
COPD = chronic obstructive pulmonary disease
CRP = human c-reactive protein
CYMA: a metabolite of acrylonitrile (VOC)
TSNA = Tobacco-specific *N*-nitrosamines
PAH = polycyclic aromatic hydrocarbons
GTT = glucose tolerance test
HbA1c = glycosylated haemoglobin
HOMA-IR = homeostatic model assessment of insulin resistance
HPMA: N-acetyl-S-3-hydroxypropylcysteine, a metabolite of acrolein (VOC)
MHB3, a metabolite of 1,3-butadiene (VOC)
MU = *trans,trans*-Muconic acid (VOC, benzene)
NNAL = 4-(methylnitrosamino)-1-(3-pyridyl)-1-butanol, the principal metabolite of the lung carcinogen NNK (TSNA)
NNN = N0-nitrosonornicotine (TSNA)
NRT=nicotine replacement therapy
PHEMA= N-Acetyl-S-(1 and 2-phenyl-2-hydroxyethyl)-L-cysteine (VOC, styrine)
PMA= benzene
SIGN. = significant/significance
VOC= Volatile organic compounds

Conflict of interest:

- Flacco: The sixth year was partially supported by British American Tobacco. Two years of follow-up were funded by a research grant from the University of Catania and through crowdfunding (Kickstarter project titled “E-cigarette long-term efficacy and safety: a study to complete”). Besides 7 authors (MEF, RS, MRG, GL, MFi, PV, CM) and 7 anonymous contributors, who donated a total of €565 and €80, respectively, all other contributors were private citizens who had no role in any phase of the study.
- Goniewicz: M.L.G receives fees for serving on an advisory board from Johnson & Johnson and grant support from Pfizer.
- Hedman: LH reported grants from the Swedish Heart-Lung Foundation, the Swedish Research Council, a regional agreement between Umea University and Vasterbotten County Council (ALF), the Swedish Asthma-Allergy Foundation, VISARE NORR Fund: Northern County Councils’ Regional Federation, and Norrbotten County Council during the conduct of the study. HB reported grants from the Swedish Heart-Lung Foundation, VBG Group’s Herman Krefting Foundation for Asthma and Allergy Research, a regional agreement between Umea University and Vasterbotten County Council (ALF), Norrbotten County Council, and VISARE NORR Fund: Northern County Councils’ Regional Federation during the conduct of the study; and personal fees from Boehringer Ingelheim outside the submitted work. AL reported personal fees from AstraZeneca, Boehringer Ingelheim, Novartis, and ActiveCare outside the submitted work. ER reported grants from the Swedish Heart-Lung Foundation, Norrbotten County Council, and Umea University during the conduct of the study; and grants from FORMAS, AstraZeneca, and GlaxoSmithKline outside the submitted work. LE reported grants from VBG Group’s Herman Krefting Foundation for Asthma and Allergy Research during the conduct of the study; and grants from VBG Group’s Herman Krefting Foundation for Asthma and Allergy Research outside the submitted work.
- Li: MLG received a research grant from Pfizer and served as a member of advisory board to Johnson & Johnson, manufacturers of smoking cessation medications.
- McRobbie: H. McRobbie is Clinical Director at The Dragon Institute The Dragon Institute; reports receiving commercial research grant from Pfizer; and has received speakers bureau honoraria from Johnson&Johnson and Pfizer. M.L. Goniewicz reports receiving commercial research grant from Pfizer. P. Hajek has received speakers bureau honoraria from and is a consultant/advisory board member for the manufacturers of stop-smoking medications.
- Miller: M.L.G. has received a research grant from Pfizer, Inc. and served as a member of the scientific advisory board to Johnson & Johnson.
- Osei 2019: declared funding from public health and NGO funds, such as American Heart Association. No personal fees declared.
- Osei 2020: DeFilippis has unrelated research funding from Astra Zeneca and is a consultant on an unrelated topic for Radiometer America, Inc. No
- Polosa: in some publication Polosa declares no COI. He has founded the Center of Excellence for the Acceleration of Harm Reduction (CoEHAR), an interdepartmental Research Centre within the University of Catania. This has received significant funding from a tobacco-industry front group, the Foundation for a Smoke-Free World (Philip Morris). He is also a Special Scientific Advisor to the International Network of Nicotine Consumer Organizations (INNCO). The INNCO was formed in 2016 and represents organisations promoting Next Generation Products such as e-cigarettes, heated tobacco products and snus. In November 2018 INNCO was granted $100,300 from the Foundation for a Smoke-Free World (Philip Morris).

- Prokopowicz 2019: AP, MS–C, PO, and AS are employees of the Institute of Occupational Medicine and Environmental Health. One of the institute's objectives is outsourcing for the industrial sector, including manufacturers of e-cigarettes. AS accepted personal fees from the eSmoking Institute in Poznan, Poland, and nonfinancial support from Chic Group LTD, a manufacturer of electronic cigarettes in Poland, outside of the submitted work. LK works as an expert for the Polish National Committee for Standardization and for the European Committee for the standardization of requirements and test methods for e-liquids and emissions.
- Prokopowisz 2020: same as 2019.
- Kosmider: employees of the Institute of Occupational Medicine and Environmental Health. Leon Kosmider also works as an expert for the Polish National Committee for Standardization and for the European Committee for Standardization (CEN) in the field of requirements and test methods for e–liquids and emissions.
- Pulvers: Benowitz is a consultant to pharmaceutical companies that market smoking cessation medications and has been an expert witness in litigation against tobacco companies. The other authors have no conflicts of interest.
- Riehm: APS received an honorarium from Springer Nature Switzerland AG for Guest Editing a Special Issue of Current Sleep Medicine Reports.
- Rubinstein: Dr Benowitz is a consultant to several pharmaceutical companies that market medications to aid smoking cessation and has served as a paid expert witness in litigation against tobacco companies. Drs Ramo and Rubinstein have consulted for Carrot Inc, which makes a tobacco cessation device.
- Shahab: Dr. Shahab reports grants from Cancer Research UK during the conduct of the study and grants from Pfizer (unrestricted research funding to study smoking cessation) and personal fees from Atlantis Health Care outside of the submitted work. Dr. Goniewicz reports grants from Pfizer (2011 GRAND [Global Research Awards for Nicotine Dependence] recipient) and personal fees from Johnson & Johnson (as a member of the advisory board) outside the submitted work. Dr. Brown reports grants (unrestricted research funding to study smoking cessation) from Pfizer outside the submitted work. Dr. West reports grants, personal fees, and nonfinancial support (that is, research grants, consultancy, travel, and hospitality) from Pfizer, Johnson & Johnson, and GlaxoSmithKline outside the submitted work
- Smith: M.L.G. received a research grant from Pfizer and served as a member of an advisory board to Johnson & Johnson, pharmaceutical companies that both market smoking cessation medications. L.S. has received honoraria for talks, an unrestricted research grant and travel expenses to attend meetings and workshops by pharmaceutical companies that make smoking cessation products (Pfizer, Johnson & Johnson), has acted as a paid reviewer for grant awarding bodies and as a paid consultant for healthcare companies.
- Soule: E.S. is named on a patent application for a smartphone app that determines electronic cigarette device and liquid characteristics.
- Wang J.B.: The study was partially supported by 24K Data (http://24Kdata.com). There are no patents, products in development or marketed products to declare.
- Wiernik: GA has received speaker and consulting fees from Lundbeck and Pfizer, outside the submitted work. FL has received speaker and consulting fees from AstraZeneca, Eutherapie-Servier, Janssen, Lundbeck, Otsuka Pharmaceuticals France and Roche, outside the submitted work. CL reports grants, personal fees and non-financial support from Lundbeck, personal fees from Servier, Daiichi-Sankyo and Janssen, non-financial support from Otsuka Pharmaceuticals, outside the submitted work.

References

1. Akinkugbe AA. Cigarettes, E-cigarettes, and Adolescents' Oral Health: Findings from the Population Assessment of Tobacco and Health (PATH) Study. JDR Clin Trans Res. 2019;4(3):276-83.
2. Bhatta DN, Glantz SA. Association of E-Cigarette Use With Respiratory Disease Among Adults: A Longitudinal Analysis. Am J Prev Med. 2020;58(2):182-90.
3. Cardenas VM, Cen R, Clemens MM, Moody HL, Ekanem US, Policherla A, et al. Use of Electronic Nicotine Delivery Systems (ENDS) by pregnant women I: Risk of small-for-gestational-age birth. Tobacco induced diseases. 2019;17:44.
4. Carroll DM, Wagener TL, Peck JD, Brame LS, Thompson DM, Stephens LD, et al. Biomarkers of Exposure in ENDS Users, Smokers, and Dual Users of American Indian Descent. Tobacco regulatory science. 2018;4(2):3-15.
5. Chen DTH, Kyriakos CN. Cigarette and e-cigarettes dual users, exclusive users and covid-19: Findings from four uk birth cohort studies. International Journal of Environmental Research and Public Health. 2021;18(8).
6. Cho JH, Paik SY. Association between Electronic Cigarette Use and Asthma among High School Students in South Korea. Plos One. 2016;11(3):13.

7. Choi DW, Jeon J, Lee SA, Han KT, Park EC, Jang SI. Association between Smoking Behavior Patterns and Glycated Hemoglobin Levels in a General Population. International journal of environmental research and public health. 2018;15(10).
8. Chung SJ, Kim B-K, Oh JH, Shim J-S, Chang Y-S, Cho S-H, et al. Novel tobacco products including electronic cigarette and heated tobacco products increase risk of allergiv rhinitis and asthma in adolescents: Analysis of Korean youth survey. Allergy. 2020;75:9.
9. Clemens MM, Cardenas VM, Fischbach LA, Cen R, Siegel ER, Eswaran H, et al. Use of electronic nicotine delivery systems by pregnant women II: Hair biomarkers for exposures to nicotine and tobacco-specific nitrosamines. Tobacco induced diseases. 2019;17:50.
10. Dinkeloo E, Grier TL, Brooks RD, Jones BH. Vaping, Smoking, and the Physical Fitness of Active Young Men. Am J Prev Med. 2020;58(1):e31-e7.
11. Fetterman JL, Keith RJ, Palmisano JN, McGlasson KL, Weisbrod RM, Majid S, et al. Alterations in Vascular Function Associated With the Use of Combustible and Electronic Cigarettes. Journal of the American Heart Association. 2020;9(9):e014570.
12. Flacco ME, Ferrante M, Fiore M, Marzuillo C, La Vecchia C, Gualano MR, et al. Cohort study of electronic cigarette use: safety and effectiveness after 4 years of follow-up. Eur Rev Med Pharmacol Sci. 2019;23(1):402-12.
13. Flacco ME, Fiore M, Acuti Martellucci C, Ferrante M, Gualano MR, Liguori G, et al. Tobacco vs. electronic cigarettes: absence of harm reduction after six years of follow-up. European review for medical and pharmacological sciences. 2020;24(7):3923-34.
14. Gaiha SM, Cheng J, Halpern-Felsher B. Association Between Youth Smoking, Electronic Cigarette Use, and COVID-19. J Adolesc Health. 2020;67(4):519-23.
15. Goniewicz ML, Smith DM, Edwards KC, Blount BC, Caldwell KL, Feng J, et al. Comparison of Nicotine and Toxicant Exposure in Users of Electronic Cigarettes and Combustible Cigarettes. JAMA Netw Open. 2018;1(8):e185937.
16. Harlow AF, Hatch EE, Wesselink AK, Rothman KJ, Wise LA. E-cigarettes and Fecundability: Results from a Prospective Preconception Cohort Study. Am J Epidemiol. 2020.
17. Hedman L, Backman H, Stridsman C, Bosson JA, Lundbäck M, Lindberg A, et al. Association of Electronic Cigarette Use With Smoking Habits, Demographic Factors, and Respiratory Symptoms. JAMA Netw Open. 2018;1(3):e180789.
18. Jain RB. Concentrations of cadmium, lead, and mercury in blood among US cigarettes, cigars, electronic cigarettes, and dual cigarette-e-cigarette users. Environ Pollut. 2019;251:970-4.
19. Keith RJ, Fetterman JL, Orimoloye OA, Dardari Z, Lorkiewicz PK, Hamburg NM, et al. Characterization of Volatile Organic Compound Metabolites in Cigarette Smokers, Electronic Nicotine Device Users, Dual Users, and Nonusers of Tobacco. Nicotine Tob Res. 2020;22(2):264-72.
20. Kim CY, Paek YJ, Seo HG, Cheong YS, Lee CM, Park SM, et al. Dual use of electronic and conventional cigarettes is associated with higher cardiovascular risk factors in Korean men. Sci Rep. 2020;10(1):5612.
21. Kim T, Choi H, Kang J, Kim J. Association between electronic cigarette use and metabolic syndrome in the Korean general population: A nationwide population-based study. PLoS ONE. 2020;15(8 August 2020).
22. Kim T, Kim Y, Kang J. Association of electronic cigarette exposure with serum uric acid level and hyperuricemia: 2016-2017 Korea National Health and Nutritional Examination Survey. Plos One. 2021;16(3).
23. Leavens ELS, Ford BR, Ojo-Fati O, Winkelman TNA, Vickery KD, Japuntich SJ, et al. Electronic cigarette use patterns and chronic health conditions among people experiencing homelessness in MN: a statewide survey. BMC Public Health. 2020;20(1):1-8.

24. Lee A, Lee SY, Lee K-S. The Use of Heated Tobacco Products is Associated with Asthma, Allergiv Rhinitis, and Atopic Dermatitis in Korean Adolescents Scientific reports. 2019;9:8.
25. Li D, Sundar IK, McIntosh S, Ossip DJ, Goniewicz ML, O'Connor RJ, et al. Association of smoking and electronic cigarette use with wheezing and related respiratory symptoms in adults: cross-sectional results from the Population Assessment of Tobacco and Health (PATH) study, wave 2. Tob Control. 2020;29(2):140-7.
26. Mainous AG, 3rd, Yadav S, Hong YR, Huo J. e-Cigarette and Conventional Tobacco Cigarette Use, Dual Use, and C-Reactive Protein. J Am Coll Cardiol. 2020;75(17):2271-3.
27. Manzoli L, Flacco ME, Fiore M, La Vecchia C, Marzuillo C, Gualano MR, et al. Electronic Cigarettes Efficacy and Safety at 12 Months: Cohort Study. PLoS One. 2015;10(6):e0129443.
28. Manzoli L, Flacco ME, Ferrante M, La Vecchia C, Siliquini R, Ricciardi W, et al. Cohort study of electronic cigarette use: effectiveness and safety at 24 months. Tob Control. 2017;26(3):284-92.
29. McDonnell BP, Dicker P, Regan CL, McDonnell BP, Regan CL. Electronic cigarettes and obstetric outcomes: a prospective observational study. BJOG: An International Journal of Obstetrics & Gynaecology. 2020;127(6):750-6.
30. McRobbie H, Phillips A, Goniewicz ML, Smith KM, Knight-West O, Przulj D, et al. Effects of Switching to Electronic Cigarettes with and without Concurrent Smoking on Exposure to Nicotine, Carbon Monoxide, and Acrolein. Cancer prevention research (Philadelphia, Pa). 2015;8(9):873-8.
31. Merianos AL, Jandarov RA, Choi K, Fiser KA, Mahabee-Gittens EM. Combustible and electronic cigarette use and insufficient sleep among U.S. high school students. Prev Med. 2021:106505.
32. Miller CR, Shi H, Li D, Goniewicz ML. Cross-Sectional Associations of Smoking and E-cigarette Use with Self-Reported Diagnosed Hypertension: Findings from Wave 3 of the Population Assessment of Tobacco and Health Study. Toxics. 2021;9(52):14.
33. Orimoloye OA, Uddin SMI, Chen LC, Osei AD, Mirbolouk M, Malovichko MV, et al. Electronic cigarettes and insulin resistance in animals and humans: Results of a controlled animal study and the National Health and Nutrition Examination Survey (NHANES 2013-2016). PLoS One. 2019;14(12):e0226744.
34. Osei AD, Mirbolouk M, Orimoloye OA, Dzaye O, Uddin SMI, Benjamin EJ, et al. Association Between E-Cigarette Use and Cardiovascular Disease Among Never and Current Combustible-Cigarette Smokers. Am J Med. 2019;132(8):949-54.e2.
35. Osei AD, Mirbolouk M, Orimoloye OA, Dzaye O, Uddin SMI, Benjamin EJ, et al. Association Between E-Cigarette Use and Chronic Obstructive Pulmonary Disease by Smoking Status: Behavioral Risk Factor Surveillance System 2016 and 2017. Am J Prev Med. 2020;58(3):336-42.
36. Parekh T, Pemmasani S, Desai R. Risk of Stroke With E-Cigarette and Combustible Cigarette Use in Young Adults. American journal of preventive medicine. 2019;28.
37. Parekh T, Owens C, Fay K, Phillips J, Kitsantas P. Use of e-Cigarettes and Development of Respiratory Conditions in Women of Childbearing Age. South Med J. 2020;113(10):488-94.
38. Piper ME, Baker TB, Benowitz NL, Kobinsky KH, Jorenby DE. Dual Users Compared to Smokers: Demographics, Dependence, and Biomarkers. Nicotine Tob Res. 2019;21(9):1279-84.
39. Prokopowicz A, Sobczak A, Szuła-Chraplewska M, Ochota P, Kośmider L. Exposure to Cadmium and Lead in Cigarette Smokers Who Switched to Electronic Cigarettes. Nicotine Tob Res. 2019;21(9):1198-205.

40. Prokopowicz A, Sobczak A, Szdzuj J, Grygoyć K, Kośmider L. Metal Concentration Assessment in the Urine of Cigarette Smokers Who Switched to Electronic Cigarettes: A Pilot Study. Int J Environ Res Public Health. 2020;17(6).
41. Riehm KE, Rojo-Wissar DM, Feder KA, Mojtabai R, Spira AP, Thrul J, et al. E-cigarette use and sleep-related complaints among youth. J Adolesc. 2019;76:48-54.
42. Rostron BL, Corey CG, Chang JT, van Bemmel DM, Miller ME, Chang CM. Associations of Cigarettes Smoked Per Day with Biomarkers of Exposure Among U.S. Adult Cigarette Smokers in the Population Assessment of Tobacco and Health (PATH) Study Wave 1 (2013-2014). Cancer Epidemiol Biomarkers Prev. 2019;28(9):1443-53.
43. Sanou AZ, Ziadeh C, Stahlman S, Clausen SS. Acute Respiratory Infections Among Active Component Service Members Who Use Combustible Tobacco Products and/or E-cigarette/Vaping Products, U.S. Armed Forces, 2018-2019. Msmr. 2020;27(11):2-7.
44. Shahab L, Goniewicz ML, Blount BC, Brown J, McNeill A, Alwis KU, et al. Nicotine, Carcinogen, and Toxin Exposure in Long-Term E-Cigarette and Nicotine Replacement Therapy Users: A Cross-sectional Study. Ann Intern Med. 2017;166(6):390-400.
45. Smith DM, Shahab L, Blount BC, Gawron M, Kosminder L, Sobczak A, et al. Differences in Exposure to Nicotine, Tobacco-Specific Nitrosamines, and Volatile Organic Compounds among Electronic Cigarette Users, Tobacco Smokers, and Dual Users from Three Countries. Toxics. 2020;8(4).
46. Vindhyal MR, Okut H, Ablah E, Ndunda PM, Kallail KJ, Choi WS. Cardiovascular Outcomes Associated With Adult Electronic Cigarette Use. Cureus. 2020;12(8):e9618.
47. Wang JB, Olgin JE, Nah G, Vittinghoff E, Cataldo JK, Pletcher MJ, et al. Cigarette and e-cigarette dual use and risk of cardiopulmonary symptoms in the Health eHeart Study. PLoS One. 2018;13(7):e0198681.
48. Wang X, Lee NL, Burstyn I. Smoking and use of electronic cigarettes (vaping) in relation to preterm birth and smal-for-gestational-age in a 2016 U.S. national sample. Preventive Medicine. 2020;134:8.
49. Wills TA, Pagano I, Williams R, Tam E. E-cigarette use and respiratory disorder in an adults sample. Drug Alcohol Depend. 2019;194:8.
50. Wills TA, Choi K, Pagano I. E-cigarette Use Associated With Asthma Independent of Cigarette Smoking and Marijuana in a 2017 National Sample of Adolescents. Journal of Adolescent Health. 2020;67:7.
51. Xie Z, Ossip DJ, Rahman I, Li D. Use of Electronic Cigarettes and Self-Reported Chronic Obstructive Pulmonary Disease Diagnosis in Adults. Nicotine Tob Res. 2020;22(7):1155-61.
52. Ye D, Gajendra S, Lawyer G, Jadeja N, Pishey D, Pathagunti S, et al. Inflammatory biomarkers and growth factors in saliva and gingival crevicular fluid of e-cigarette users, cigarette smokers, and dual smokers: A pilot study. J Periodontol. 2020.

Appendix S1 **JBI CRITICAL APPRAISAL CHECKLIST FOR COHORT STUDIES**

Author____________McRobbie H________________________ Year___2015______

		Yes	No	Unclear	Not applicable
1.	Were the two groups similar and recruited from the same population?	X	☐	☐	☐
2.	Were the exposures measured similarly to assign people to both exposed and unexposed groups?	X	☐	☐	☐
3.	Was the exposure measured in a valid and reliable way?	☐	X	☐	☐
4.	Were confounding factors identified?	☐	X	☐	☐
5.	Were strategies to deal with confounding factors stated?	☐	X	☐	☐
6.	Were the groups/participants free of the outcome at the start of the study (or at the moment of exposure)?	X	☐	☐	☐
7.	Were the outcomes measured in a valid and reliable way?	X	☐	☐	☐
8.	Was the follow up time reported and sufficient to be long enough for outcomes to occur?	X	☐	☐	☐
9.	Was follow up complete, and if not, were the reasons to loss to follow up described and explored?	☐	X	☐	☐
10.	Were strategies to address incomplete follow up utilized?	☐	X	☐	☐
11.	Was appropriate statistical analysis used?	X	☐	☐	☐

NOT ADJUSTED FOR FORMER TOBACCO CONSUMPTION

JBI CRITICAL APPRAISAL CHECKLIST FOR COHORT STUDIES

Author________________________Bhatta DN____________________Year___2020______

		Yes	No	Unclear	Not applicable
1.	Were the two groups similar and recruited from the same population?	X	☐	☐	☐
2.	Were the exposures measured similarly to assign people to both exposed and unexposed groups?	X	☐	☐	☐
3.	Was the exposure measured in a valid and reliable way?	☐	X	☐	☐
4.	Were confounding factors identified?	X	☐	☐	☐
5.	Were strategies to deal with confounding factors stated?	X	☐	☐	☐
6.	Were the groups/participants free of the outcome at the start of the study (or at the moment of exposure)?	X	☐	☐	☐
7.	Were the outcomes measured in a valid and reliable way?	X	☐	☐	☐
8.	Was the follow up time reported and sufficient to be long enough for outcomes to occur?	X	☐	☐	☐
9.	Was follow up complete, and if not, were the reasons to loss to follow up described and explored?	X	☐	☐	☐
10.	Were strategies to address incomplete follow up utilized?	X	☐	☐	☐
11.	Was appropriate statistical analysis used?	X	☐	☐	☐

NOT ADJUSTED FOR FORMER TOBACCO CONSUMPTION

JBI CRITICAL APPRAISAL CHECKLIST FOR COHORT STUDIES

Author_____________ ________Sanou AZ__________________ Year___2020______

		Yes	No	Unclear	Not applicable
1.	Were the two groups similar and recruited from the same population?	X	☐	☐	☐
2.	Were the exposures measured similarly to assign people to both exposed and unexposed groups?	X	☐	☐	☐
3.	Was the exposure measured in a valid and reliable way?	☐	X	☐	☐
4.	Were confounding factors identified?	X	☐	☐	☐
5.	Were strategies to deal with confounding factors stated?	X	☐	☐	☐
6.	Were the groups/participants free of the outcome at the start of the study (or at the moment of exposure)?	X	☐	☐	☐
7.	Were the outcomes measured in a valid and reliable way?	X	☐	☐	☐
8.	Was the follow up time reported and sufficient to be long enough for outcomes to occur?	X	☐	☐	☐
9.	Was follow up complete, and if not, were the reasons to loss to follow up described and explored?	☐	☐	X	☐
10.	Were strategies to address incomplete follow up utilized?	☐	☐	X	☐
11.	Was appropriate statistical analysis used?	X	☐	☐	☐

NOT ADJUSTED FOR FORMER TOBACCO CONSUMPTION

JBI CRITICAL APPRAISAL CHECKLIST FOR COHORT STUDIES

Author_____________Cardenas VM_____________________Year____2020_____

		Yes	No	Unclear	Not applicable
1.	Were the two groups similar and recruited from the same population?	X	☐	☐	☐
2.	Were the exposures measured similarly to assign people to both exposed and unexposed groups?	X	☐	☐	☐
3.	Was the exposure measured in a valid and reliable way?	☐	X	☐	☐
4.	Were confounding factors identified?	X	☐	☐	☐
5.	Were strategies to deal with confounding factors stated?	X	☐	☐	☐
6.	Were the groups/participants free of the outcome at the start of the study (or at the moment of exposure)?	☐	☐	☐	X
7.	Were the outcomes measured in a valid and reliable way?	X	☐	☐	☐
8.	Was the follow up time reported and sufficient to be long enough for outcomes to occur?	X	☐	☐	☐
9.	Was follow up complete, and if not, were the reasons to loss to follow up described and explored?	X	☐	☐	☐
10.	Were strategies to address incomplete follow up utilized?	☐	☐	☐	X
11.	Was appropriate statistical analysis used?	X	☐	☐	☐

NOT ADJUSTED FOR FORMER TOBACCO CONSUMPTION

JBI CRITICAL APPRAISAL CHECKLIST FOR COHORT STUDIES

Author____________Clemens MM____________________Year___2019______

		Yes	No	Unclear	Not applicable
1.	Were the two groups similar and recruited from the same population?	X	☐	☐	☐
2.	Were the exposures measured similarly to assign people to both exposed and unexposed groups?	X	☐	☐	☐
3.	Was the exposure measured in a valid and reliable way?	☐	X	☐	☐
4.	Were confounding factors identified?	X	☐	☐	☐
5.	Were strategies to deal with confounding factors stated?	X	☐	☐	☐
6.	Were the groups/participants free of the outcome at the start of the study (or at the moment of exposure)?	☐	☐	☐	X
7.	Were the outcomes measured in a valid and reliable way?	X	☐	☐	☐
8.	Was the follow up time reported and sufficient to be long enough for outcomes to occur?	X	☐	☐	☐
9.	Was follow up complete, and if not, were the reasons to loss to follow up described and explored?	X	☐	☐	☐
10.	Were strategies to address incomplete follow up utilized?	☐	☐	☐	X
11.	Was appropriate statistical analysis used?	X	☐	☐	☐

NOT ADJUSTED FOR FORMER TOBACCO CONSUMPTION

JBI CRITICAL APPRAISAL CHECKLIST FOR COHORT STUDIES

Author_____________Flacco ME___________________Year__2020_______

		Yes	No	Unclear	Not applicable
1.	Were the two groups similar and recruited from the same population?	X	☐	☐	☐
2.	Were the exposures measured similarly to assign people to both exposed and unexposed groups?	X	☐	☐	☐
3.	Was the exposure measured in a valid and reliable way?	☐	X	☐	☐
4.	Were confounding factors identified?	X	☐	☐	☐
5.	Were strategies to deal with confounding factors stated?	X	☐	☐	☐
6.	Were the groups/participants free of the outcome at the start of the study (or at the moment of exposure)?	X	☐	☐	☐
7.	Were the outcomes measured in a valid and reliable way?	X	☐	☐	☐
8.	Was the follow up time reported and sufficient to be long enough for outcomes to occur?	X	☐	☐	☐
9.	Was follow up complete, and if not, were the reasons to loss to follow up described and explored?	☐	X	☐	☐
10.	Were strategies to address incomplete follow up utilized?	X	☐	☐	☐
11.	Was appropriate statistical analysis used?	X	☐	☐	☐

JBI CRITICAL APPRAISAL CHECKLIST FOR COHORT STUDIES

Author___________Harlow A_____________________ Year_____2020____

		Yes	No	Unclear	Not applicable
1.	Were the two groups similar and recruited from the same population?	X	☐	☐	☐
2.	Were the exposures measured similarly to assign people to both exposed and unexposed groups?	X	☐	☐	☐
3.	Was the exposure measured in a valid and reliable way?	☐	X	☐	☐
4.	Were confounding factors identified?	X	☐	☐	☐
5.	Were strategies to deal with confounding factors stated?	X	☐	☐	☐
6.	Were the groups/participants free of the outcome at the start of the study (or at the moment of exposure)?	☐	☐	☐	X
7.	Were the outcomes measured in a valid and reliable way?	X	☐	☐	☐
8.	Was the follow up time reported and sufficient to be long enough for outcomes to occur?	X	☐	☐	☐
9.	Was follow up complete, and if not, were the reasons to loss to follow up described and explored?	X	☐	☐	☐
10.	Were strategies to address incomplete follow up utilized?	☐	☐	X	☐
11.	Was appropriate statistical analysis used?	X	☐	☐	☐

JBI CRITICAL APPRAISAL CHECKLIST FOR COHORT STUDIES

Author_______________________Manzoli L_____________ Year__2015_______

		Yes	No	Unclear	Not applicable
1.	Were the two groups similar and recruited from the same population?	X	☐	☐	☐
2.	Were the exposures measured similarly to assign people to both exposed and unexposed groups?	X	☐	☐	☐
3.	Was the exposure measured in a valid and reliable way?	☐	X	☐	☐
4.	Were confounding factors identified?	X	☐	☐	☐
5.	Were strategies to deal with confounding factors stated?	X	☐	☐	☐
6.	Were the groups/participants free of the outcome at the start of the study (or at the moment of exposure)?	X	☐	☐	☐
7.	Were the outcomes measured in a valid and reliable way?	X	☐	☐	☐
8.	Was the follow up time reported and sufficient to be long enough for outcomes to occur?	X	☐	☐	☐
9.	Was follow up complete, and if not, were the reasons to loss to follow up described and explored?	☐	X	☐	☐
10.	Were strategies to address incomplete follow up utilized?	☐	X	☐	☐
11.	Was appropriate statistical analysis used?	X	☐	☐	☐

JBI CRITICAL APPRAISAL CHECKLIST FOR COHORT STUDIES

Author________________________Manzoli L____________ Year____2017_____

		Yes	No	Unclear	Not applicable
1.	Were the two groups similar and recruited from the same population?	X	☐	☐	☐
2.	Were the exposures measured similarly to assign people to both exposed and unexposed groups?	X	☐	☐	☐
3.	Was the exposure measured in a valid and reliable way?	☐	X	☐	☐
4.	Were confounding factors identified?	X	☐	☐	☐
5.	Were strategies to deal with confounding factors stated?	X	☐	☐	☐
6.	Were the groups/participants free of the outcome at the start of the study (or at the moment of exposure)?	X	☐	☐	☐
7.	Were the outcomes measured in a valid and reliable way?	X	☐	☐	☐
8.	Was the follow up time reported and sufficient to be long enough for outcomes to occur?	X	☐	☐	☐
9.	Was follow up complete, and if not, were the reasons to loss to follow up described and explored?	☐	X	☐	☐
10.	Were strategies to address incomplete follow up utilized?	☐	X	☐	☐
11.	Was appropriate statistical analysis used?	X	☐	☐	☐

JBI CRITICAL APPRAISAL CHECKLIST FOR COHORT STUDIES

Author____________ ____McDonnell BP___________________ Year___2020______

		Yes	No	Unclear	Not applicable
1.	Were the two groups similar and recruited from the same population?	X	☐	☐	☐
2.	Were the exposures measured similarly to assign people to both exposed and unexposed groups?	X	☐	☐	☐
3.	Was the exposure measured in a valid and reliable way?	☐	☐	X	☐
4.	Were confounding factors identified?	X	☐	☐	☐
5.	Were strategies to deal with confounding factors stated?	X	☐	☐	☐
6.	Were the groups/participants free of the outcome at the start of the study (or at the moment of exposure)?	☐	☐	☐	X
7.	Were the outcomes measured in a valid and reliable way?	X	☐	☐	☐
8.	Was the follow up time reported and sufficient to be long enough for outcomes to occur?	X	☐	☐	☐
9.	Was follow up complete, and if not, were the reasons to loss to follow up described and explored?	X	☐	☐	☐
10.	Were strategies to address incomplete follow up utilized?	☐	☐	☐	X
11.	Was appropriate statistical analysis used?	X	☐	☐	☐

NOT ADJUSTED FOR FORMER TOBACCO CONSUMPTION

JBI CRITICAL APPRAISAL CHECKLIST FOR COHORT STUDIES

Author_______________Riehm KE_________________ Year___2019______

		Yes	No	Unclear	Not applicable
1.	Were the two groups similar and recruited from the same population?	X	☐	☐	☐
2.	Were the exposures measured similarly to assign people to both exposed and unexposed groups?	X	☐	☐	☐
3.	Was the exposure measured in a valid and reliable way?	☐	X	☐	☐
4.	Were confounding factors identified?	X	☐	☐	☐
5.	Were strategies to deal with confounding factors stated?	X	☐	☐	☐
6.	Were the groups/participants free of the outcome at the start of the study (or at the moment of exposure)?	☐	☐	☐	X
7.	Were the outcomes measured in a valid and reliable way?	X	☐	☐	☐
8.	Was the follow up time reported and sufficient to be long enough for outcomes to occur?	X	☐	☐	☐
9.	Was follow up complete, and if not, were the reasons to loss to follow up described and explored?	X	☐	☐	☐
10.	Were strategies to address incomplete follow up utilized?	☐	☐	☐	X
11.	Was appropriate statistical analysis used?	X	☐	☐	☐

NOT ADJUSTED FOR FORMER TOBACCO CONSUMPTION

JBI CRITICAL APPRAISAL CHECKLIST FOR COHORT STUDIES

Author________________Wang X__________________Year___2020______

		Yes	No	Unclear	Not applicable
1.	Were the two groups similar and recruited from the same population?	X	☐	☐	☐
2.	Were the exposures measured similarly to assign people to both exposed and unexposed groups?	X	☐	☐	☐
3.	Was the exposure measured in a valid and reliable way?	☐	X	☐	☐
4.	Were confounding factors identified?	X	☐	☐	☐
5.	Were strategies to deal with confounding factors stated?	X	☐	☐	☐
6.	Were the groups/participants free of the outcome at the start of the study (or at the moment of exposure)?	☐	☐	☐	X
7.	Were the outcomes measured in a valid and reliable way?	X	☐	☐	☐
8.	Was the follow up time reported and sufficient to be long enough for outcomes to occur?	X	☐	☐	☐
9.	Was follow up complete, and if not, were the reasons to loss to follow up described and explored?	X	☐	☐	☐
10.	Were strategies to address incomplete follow up utilized?	☐	☐	☐	X
11.	Was appropriate statistical analysis used?	X	☐	☐	☐

NOT ADJUSTED FOR FORMER TOBACCO CONSUMPTION

Appendix S2. Effect measures, data items, study design, characteristics of studies, definition of use and prevalence of use

Effect measures

Effect measures varied depending on the outcome. Most papers on symptoms or disease risk presented unadjusted and adjusted odds ratios with a 95% confidence interval (CI). We present adjusted odds ratios if available (aCI). Papers on toxic effects typically presented geometric means and 95% CI and/or ranges and interquartile intervals.

Data items

Papers were included if any outcome data comparing DU with ESCC were presented, even if significance levels between ESCC and DU were not shown. We extracted the same predefined information from all papers (Appendix 3). If data on any variable were missing, we searched in supplementary material and/or in study protocols.

Study design

One study was a switch-of-product experimental studies [1]. The majority of studies reported results of cross-sectional analyses, mostly using self-reported data from large population-based surveys. Thirteen papers/10 studies reported results from studies with a prospective design [1-13].

Almost all population-based studies had weighted data for non-response, thereby diminishing the risk of selection bias. One study adjusted for only one confounder [14], while most adjusted for several confounders, and some for up to 13 [3, 8, 9, 15]. The experimental human studies that recruited volunteers in newspapers [1], print or radio, in EC shops, social media, general practice, etc. [3, 4, 8, 9, 16], or where the recruitment method was not defined [17] were assessed to have a high risk of selection bias. Studies with pregnant women/women trying to conceive [5, 6] and military personnel [7, 11, 18] were supposed to have a high risk of social desirability bias (underreporting of EC/CC use). Studies investigating symptoms/disease in the past were at risk of recall bias.

Characteristics of studies (Appendix S3)

Fifty-two studies were included: three from the UK [1, 19, 20], one from Ireland [2], one from Sweden [21], two from Poland [17], [22], four from Italy [3, 4, 8, 9], seven from South Korea [23-29], one study used data from three countries (Poland, the UK and the USA) [30], and the remaining were from the USA. Seven studies reported on health outcome in adolescents [13, 23, 25, 29, 31-33], with the age for inclusion ranging from 12 [34] to 20 years [19]; the rest included adults/young adults.

Definitions of use

There was great variation both in duration and in frequency of use of products for details. The definitions of ESCC, EC users and DUs were based on self-reports and varied a lot across studies. There was variation both in duration and in frequency of use of products. Many studies included persons with current use, defined as use in the past 30 days (DUs = past 30 days use of both CCs and of ECs) [6, 11, 18, 23, 25, 32, 33, 35-37], some included current use as in past 6 months [3, 8, 9, 20] [30] or use in past 12 months [13]. A study including adolescents defined users as ever users [29]. Several studies defined smokers and EC users differently, and some had complex definitions (Appendix 3 for details).

Between 36% [38] and 97% [28] of EC users also smoked CCs, as reported in large population-based studies including adults. Among adolescents, smoking was reported in between 29% [32] and 66% [31] of EC users. At the population level, most of the nationally representative surveys reported DU in 1–3% of the general population.

Definition of exclusive smokers and of dual users

A study including pregnant women measured use during the 3 months before their pregnancy, and during the last 3 months of pregnancy [12]. Several studies defined current use as daily and some-day use of both products [5] [39] [7] [40] [41], while some did not provide any detailed definition of current users [19] [24] [2] [16] [42]. Two studies included long-term users defined as CC use for at least 2 years and EC use for at least 6 months [17] [22]. Some included persons with use of the product during the last five days [34] [43]. In several studies smokers and EC users were defined differently, e.g. smokers were defined as those who gave an affirmative answer to the question “Do you smoke?” while EC users were those who used EC sometimes or daily [21], or smokers had smoked

every day and vapers had used EC every or some days [44], or they had used EC once a week for 3 months and smoked daily for 3 months [45]. Some studies used complex definitions of dual users such as: smoked ≥ 5 cigarettes per day (CPD) in the past 3 months, smoked in the past 24 hours, used an EC every day in the past 3 months, used an EC in the past 24 hours, and had not used any other tobacco products in the past 3 months [14], smoked more than 100 CC in their lifetime and now smoke every day or some days/currently [46] [47] [48] [49] and have used EC for the past month [26] [27] [50] [51], or those who with a lifetime usage of ≥100 cigarettes and EC and CC usage of at least 5 days per week [52].

Prevalence of dual use in the population

In South Korea large population based studies including adults found that 84% [26], 85% [27] and 97% [28] of EC users also smoked CC. In Sweden 67% of EC users in the adult general population were also smokers [21] whereas a population-based study from the UK found that only 37% of adult EC users [19] also smoked. In the population-based surveys from the USA the lowest reported prevalence of dual use in adults was 36% [38] and the highest was 77% [39]. The largest of the studies from USA, based on 2016 and 2017 Behavioral Risk Factor Surveillance System national survey data on almost one million adult participants reported that 64% had a dual use [49]. Among adolescents, smoking was reported in 29% [32], 45% [13] and 66% [31] of EC users in the USA and in 36% [25] of EC users in South Korea.

At population level most of the included nationally representative surveys reported DU in 1-3% of the general population; e.g. 1.1% in Sweden [21], 1.8% in South Korea [27], 2.2% in UK [19] and 1.8% [49], 2.9% [48] and 3% [47] in the USA. Population-based studies with the lowest rate of dual users, 0.7% [7] and 0.8% [12], were studies in pregnant women. Large population-based studies including adolescents reported DU in between 1.4% [31] (USA) and 5.6% [23] (South Korea) of the included youth. The highest rate of DU in a nationally representative survey was reported in the National Health Interview Survey (2014, 2016, 2017 and 2018 data) where 13.3% used CC and EC every day or some days [40].

References

1. McRobbie H, Phillips A, Goniewicz ML, Smith KM, Knight-West O, Przulj D, Hajek P: Effects of Switching to Electronic Cigarettes with and without Concurrent Smoking on Exposure to Nicotine, Carbon Monoxide, and Acrolein. *Cancer prevention research (Philadelphia, Pa)* 2015, 8(9):873-878.
2. McDonnell BP, Dicker P, Regan CL, McDonnell BP, Regan CL: Electronic cigarettes and obstetric outcomes: a prospective observational study. *BJOG: An International Journal of Obstetrics & Gynaecology* 2020, 127(6):750-756.
3. Flacco ME, Ferrante M, Fiore M, Marzuillo C, La Vecchia C, Gualano MR, Liguori G, Fragassi G, Carradori T, Bravi F *et al*: Cohort study of electronic cigarette use: safety and effectiveness after 4 years of follow-up. *Eur Rev Med Pharmacol Sci* 2019, 23(1):402-412.
4. Flacco ME, Fiore M, Acuti Martellucci C, Ferrante M, Gualano MR, Liguori G, Bravi F, Pirone GM, Marzuillo C, Manzoli L: Tobacco vs. electronic cigarettes: absence of harm reduction after six years of follow-up. *European review for medical and pharmacological sciences* 2020, 24(7):3923-3934.
5. Cardenas VM, Cen R, Clemens MM, Moody HL, Ekanem US, Policherla A, Fischbach LA, Eswaran H, Magann EF, Delongchamp RR *et al*: Use of Electronic Nicotine Delivery Systems (ENDS) by pregnant women I: Risk of small-for-gestational-age birth. *Tobacco induced diseases* 2019, 17:44.
6. Clemens MM, Cardenas VM, Fischbach LA, Cen R, Siegel ER, Eswaran H, Ekanem US, Policherla A, Moody HL, Magann EF *et al*: Use of electronic nicotine delivery systems by pregnant women II: Hair biomarkers for exposures to nicotine and tobacco-specific nitrosamines. *Tobacco induced diseases* 2019, 17:50.
7. Harlow AF, Hatch EE, Wesselink AK, Rothman KJ, Wise LA: E-cigarettes and Fecundability: Results from a Prospective Preconception Cohort Study. *Am J Epidemiol* 2020.
8. Manzoli L, Flacco ME, Fiore M, La Vecchia C, Marzuillo C, Gualano MR, Liguori G, Cicolini G, Capasso L, D'Amario C *et al*: Electronic Cigarettes Efficacy and Safety at 12 Months: Cohort Study. *PLoS One* 2015, 10(6):e0129443.
9. Manzoli L, Flacco ME, Ferrante M, La Vecchia C, Siliquini R, Ricciardi W, Marzuillo C, Villari P, Fiore M: Cohort study of electronic cigarette use: effectiveness and safety at 24 months. *Tob Control* 2017, 26(3):284-292.
10. Bhatta DN, Glantz SA: Association of E-Cigarette Use With Respiratory Disease Among Adults: A Longitudinal Analysis. *Am J Prev Med* 2020, 58(2):182-190.
11. Sanou AZ, Ziadeh C, Stahlman S, Clausen SS: Acute Respiratory Infections Among Active Component Service Members Who Use Combustible Tobacco Products and/or E-cigarette/Vaping Products, U.S. Armed Forces, 2018-2019. *Msmr* 2020, 27(11):2-7.
12. Wang X, Lee NL, Burstyn I: Smoking and use of electronic cigarettes (vaping) in relation to preterm birth and smal-for-gestational-age in a 2016 U.S. national sample. *Preventive Medicine* 2020, 134:8.

13. Riehm KE, Rojo-Wissar DM, Feder KA, Mojtabai R, Spira AP, Thrul J, Crum RM: E-cigarette use and sleep-related complaints among youth. *J Adolesc* 2019, 76:48-54.
14. Carroll DM, Wagener TL, Peck JD, Brame LS, Thompson DM, Stephens LD, Campbell JE, Beebe LA: Biomarkers of Exposure in ENDS Users, Smokers, and Dual Users of American Indian Descent. *Tobacco regulatory science* 2018, 4(2):3-15.
15. Parekh T, Pemmasani S, Desai R: Risk of Stroke With E-Cigarette and Combustible Cigarette Use in Young Adults. *American journal of preventive medicine* 2019, 28.
16. Ye D, Gajendra S, Lawyer G, Jadeja N, Pishey D, Pathagunti S, et al.: Inflammatory biomarkers and growth factors in saliva and gingival crevicular fluid of e-cigarette users, cigarette smokers, and dual smokers: A pilot study. *J peridontol* 2020.
17. Prokopowicz A, Sobczak A, Szuła-Chraplewska M, Ochota P, Kośmider L: Exposure to Cadmium and Lead in Cigarette Smokers Who Switched to Electronic Cigarettes. *Nicotine Tob Res* 2019, 21(9):1198-1205.
18. Dinkeloo E, Grier TL, Brooks RD, Jones BH: Vaping, Smoking, and the Physical Fitness of Active Young Men. *Am J Prev Med* 2020, 58(1):e31-e37.
19. Chen DTH, Kyriakos CN: Cigarette and e-cigarettes dual users, exclusive users and covid-19: Findings from four uk birth cohort studies. *International Journal of Environmental Research and Public Health* 2021, 18(8).
20. Shahab L, Goniewicz ML, Blount BC, Brown J, McNeill A, Alwis KU, Feng J, Wang L, West R: Nicotine, Carcinogen, and Toxin Exposure in Long-Term E-Cigarette and Nicotine Replacement Therapy Users: A Cross-sectional Study. *Ann Intern Med* 2017, 166(6):390-400.
21. Hedman L, Backman H, Stridsman C, Bosson JA, Lundbäck M, Lindberg A, Rönmark E, Ekerljung L: Association of Electronic Cigarette Use With Smoking Habits, Demographic Factors, and Respiratory Symptoms. *JAMA Netw Open* 2018, 1(3):e180789.
22. Prokopowicz A, Sobczak A, Szdzuj J, Grygoyć K, Kośmider L: Metal Concentration Assessment in the Urine of Cigarette Smokers Who Switched to Electronic Cigarettes: A Pilot Study. *Int J Environ Res Public Health* 2020, 17(6).
23. Cho JH, Paik SY: Association between Electronic Cigarette Use and Asthma among High School Students in South Korea. *Plos One* 2016, 11(3):13.
24. Choi DW, Jeon J, Lee SA, Han KT, Park EC, Jang SI: Association between Smoking Behavior Patterns and Glycated Hemoglobin Levels in a General Population. *International journal of environmental research and public health* 2018, 15(10).
25. Chung SJ, Kim B-K, Oh JH, Shim J-S, Chang Y-S, Cho S-H, Yang M-S: Novel tobacco products including electronic cigarette and heated tobacco products increase risk of allergiv rhinitis and asthma in adolescents: Analysis of Korean youth survey. *Allergy* 2020, 75:9.
26. Kim CY, Paek YJ, Seo HG, Cheong YS, Lee CM, Park SM, Park DW, Lee K: Dual use of electronic and conventional cigarettes is associated with higher cardiovascular risk factors in Korean men. *Sci Rep* 2020, 10(1):5612.
27. Kim T, Choi H, Kang J, Kim J: Association between electronic cigarette use and metabolic syndrome in the Korean general population: A nationwide population-based study. *PLoS ONE* 2020, 15(8 August 2020).
28. Kim T, Kim Y, Kang J: Association of electronic cigarette exposure with serum uric acid level and hyperuricemia: 2016-2017 Korea National Health and Nutritional Examination Survey. *Plos One* 2021, 16(3).
29. Lee A, Lee SY, Lee K-S: The Use of Heated Tobacco Products is Associated with Asthma, Allergiv Rhinitis, and Atopic Dermatitis in Korean Adolescents *Scientific reports* 2019, 9:8.

30. Smith DM, Shahab L, Blount BC, Gawron M, Kosminder L, Sobczak A, Xia B, Sosnoff CS, Goniewicz ML: Differences in Exposure to Nicotine, Tobacco-Specific Nitrosamines, and Volatile Organic Compounds among Electronic Cigarette Users, Tobacco Smokers, and Dual Users from Three Countries. *Toxics* 2020, 8(4).
31. Akinkugbe AA: Cigarettes, E-cigarettes, and Adolescents' Oral Health: Findings from the Population Assessment of Tobacco and Health (PATH) Study. *JDR Clin Trans Res* 2019, 4(3):276-283.
32. Merianos AL, Jandarov RA, Choi K, Fiser KA, Mahabee-Gittens EM: Combustible and electronic cigarette use and insufficient sleep among U.S. high school students. *Prev Med* 2021:106505.
33. Wills TA, Choi K, Pagano I: E-cigarette Use Associated With Asthma Independent of Cigarette Smoking and Marijuana in a 2017 National Sample of Adolescents. *Journal of Adolescent Health* 2020, 67:7.
34. Jain RB: Concentrations of cadmium, lead, and mercury in blood among US cigarettes, cigars, electronic cigarettes, and dual cigarette-e-cigarette users. *Environ Pollut* 2019, 251:970-974.
35. Gaiha SM, Cheng J, Halpern-Felsher B: Association Between Youth Smoking, Electronic Cigarette Use, and COVID-19. *J Adolesc Health* 2020, 67(4):519-523.
36. Keith RJ, Fetterman JL, Orimoloye OA, Dardari Z, Lorkiewicz PK, Hamburg NM, DeFilippis AP, Blaha MJ, Bhatnagar A: Characterization of Volatile Organic Compound Metabolites in Cigarette Smokers, Electronic Nicotine Device Users, Dual Users, and Nonusers of Tobacco. *Nicotine Tob Res* 2020, 22(2):264-272.
37. Leavens ELS, Ford BR, Ojo-Fati O, Winkelman TNA, Vickery KD, Japuntich SJ, Busch AM: Electronic cigarette use patterns and chronic health conditions among people experiencing homelessness in MN: a statewide survey. *BMC Public Health* 2020, 20(1):1-8.
38. Osei AD, Mirbolouk M, Orimoloye OA, Dzaye O, Uddin SMI, Benjamin EJ, Hall ME, DeFilippis AP, Bhatnagar A, Biswal SS *et al*: Association Between E-Cigarette Use and Chronic Obstructive Pulmonary Disease by Smoking Status: Behavioral Risk Factor Surveillance System 2016 and 2017. *Am J Prev Med* 2020, 58(3):336-342.
39. Goniewicz ML, Smith DM, Edwards KC, Blount BC, Caldwell KL, Feng J, Wang L, Christensen C, Ambrose B, Borek N *et al*: Comparison of Nicotine and Toxicant Exposure in Users of Electronic Cigarettes and Combustible Cigarettes. *JAMA Netw Open* 2018, 1(8):e185937.
40. Vindhyal MR, Okut H, Ablah E, Ndunda PM, Kallail KJ, Choi WS: Cardiovascular Outcomes Associated With Adult Electronic Cigarette Use. *Cureus* 2020, 12(8):e9618.
41. Wills TA, Pagano I, Williams R, Tam E: E-cigarette use and respiratory disorder in an adults sample. *Drug Alcohol Depend* 2019, 194:8.
42. Parekh T, Owens C, Fay K, Phillips J, Kitsantas P: Use of e-Cigarettes and Development of Respiratory Conditions in Women of Childbearing Age. *South Med J* 2020, 113(10):448-494.
43. Orimoloye OA, Uddin SMI, Chen LC, Osei AD, Mirbolouk M, Malovichko MV, Sithu ID, Dzaye O, Conklin DJ, Srivastava S *et al*: Electronic cigarettes and insulin resistance in animals and humans: Results of a controlled animal study and the National Health and Nutrition Examination Survey (NHANES 2013-2016). *PLoS One* 2019, 14(12):e0226744.
44. Rostron BL, Corey CG, Chang JT, van Bemmel DM, Miller ME, Chang CM: Associations of Cigarettes Smoked Per Day with Biomarkers of Exposure Among U.S. Adult Cigarette Smokers in the Population Assessment of Tobacco and Health (PATH) Study Wave 1 (2013-2014). *Cancer Epidemiol Biomarkers Prev* 2019, 28(9):1443-1453.

45. Piper ME, Baker TB, Benowitz NL, Kobinsky KH, Jorenby DE: Dual Users Compared to Smokers: Demographics, Dependence, and Biomarkers. *Nicotine Tob Res* 2019, 21(9):1279-1284.
46. Li D, Sundar IK, McIntosh S, Ossip DJ, Goniewicz ML, O'Connor RJ, Rahman I: Association of smoking and electronic cigarette use with wheezing and related respiratory symptoms in adults: cross-sectional results from the Population Assessment of Tobacco and Health (PATH) study, wave 2. *Tob Control* 2020, 29(2):140-147.
47. Miller CR, Shi H, Li D, Goniewicz ML: Cross-Sectional Associations of Smoking and E-cigarette Use with Self-Reported Diagnosed Hypertension: Findings from Wave 3 of the Population Assessment of Tobacco and Health Study. *Toxics* 2021, 9(52):14.
48. Osei AD, Mirbolouk M, Orimoloye OA, Dzaye O, Uddin SMI, Benjamin EJ, Hall ME, DeFilippis AP, Stokes A, Bhatnagar A *et al*: Association Between E-Cigarette Use and Cardiovascular Disease Among Never and Current Combustible-Cigarette Smokers. *Am J Med* 2019, 132(8):949-954.e942.
49. Xie Z, Ossip DJ, Rahman I, Li D: Use of Electronic Cigarettes and Self-Reported Chronic Obstructive Pulmonary Disease Diagnosis in Adults. *Nicotine Tob Res* 2020, 22(7):1155-1161.
50. Wang JB, Olgin JE, Nah G, Vittinghoff E, Cataldo JK, Pletcher MJ, Marcus GM: Cigarette and e-cigarette dual use and risk of cardiopulmonary symptoms in the Health eHeart Study. *PLoS One* 2018, 13(7):e0198681.
51. Mainous Ar, Yadav S, Hong Y, Huo J: E-Cigarette and Conventional Tobacco Cigarette Use, Dual Use, and C-Reactive Protein. *Journal of the American College of Cardiology* 2020, 75(17):2271-2273.
52. Fetterman JL, Keith RJ, Palmisano JN, McGlasson KL, Weisbrod RM, Majid S, Bastin R, Stathos MM, Stokes AC, Robertson RM *et al*: Alterations in Vascular Function Associated With the Use of Combustible and Electronic Cigarettes. *Journal of the American Heart Association* 2020, 9(9):e014570.
